# Early tidal despinning history recorded in the tectonics of Oz Terra, Charon

**DOI:** 10.1038/s41467-026-75069-7

**Published:** 2026-07-14

**Authors:** Hanzhang Chen, Seulgi Moon, An Yin

**Affiliations:** 1 Department of Earth, Planetary, and Space Sciences, University of California, Los Angeles, CA USA; 2 Department of Earth and Planetary Sciences, ETH, Zurich, Switzerland

**Keywords:** Tectonics, Structural geology, Geomorphology

## Abstract

Records of early geologic and thermal evolution after planetary accretion are rarely preserved on icy moons in the Solar System. Charon’s ~ 4.0 Ga surface age suggests the potential preservation of landforms induced from early orbital evolution. Here we show that despinning-induced stress inferred from compressive tectonic features explains latitudinal variations in the orientations and types of tectonic features in Charon’s northern highland, Oz Terra. In addition to global extensional features, we identify north-trending arcuate ranges in Oz Terra, interpreted as compressional in origin. Using an elastic dislocation model, we infer the geometry and kinematics of two thrust faults by fitting the observed tectonically induced topography. The inferred geometry yields a lower bound of 30–36 km for the elastic ice shell thickness at the time. The thrusts accommodate ~ 1**%** east-west compressive strain in the equatorial area. The corresponding flattening change suggests an initial rotation period of ~ 14.3 h for Charon. The modeled stress patterns also account for the east-west extensional features. Our work suggests that Charon’s surface presents an example that records the planetary despinning history, which predates the proposed global extension and cryovolcanism on Charon. The coevolution of despinning and global contraction favors a cold start for Charon, offering insights into the early thermal evolution of icy satellites in the outer Solar System.

## Abstract (web summary)

In this study, the authors show that despinning-induced stress inferred from compressive tectonic features explains latitudinal variations in the orientations and types of tectonic features in Charon’s northern highland, Oz Terra.

## Introduction

In the Voyager era, the global tectonic pattern of the planetary surface for a despun planet was predicted based on a change in its rotational state^1^. Stress from a decrease in flattening predicts north-south trending thrusts in the equatorial region, east-west trending normal faults in the polar regions, and strike-slip systems in between. Further studies incorporate thermal expansion or contraction into the model and attempt to apply the results to Mercury^2^ to explain the broader distribution of compressional tectonics. However, the optimized model still fails to match the orientations of lobate scarps on Mercury. Despite continuous efforts [e.g., ref. ^3^], no observations on planetary surfaces have yet been conclusively linked to despinning-induced tectonics since the mechanism was first proposed.

Records of early geologic and thermal evolution after planetary accretion are rarely preserved on icy moons or small bodies in the solar system. On planetary bodies with active atmospheres, such as Pluto^4^ and Titan^5^, surface processes continually reshape the landscape, erasing evidence of past tectonic features. On other bodies without current-day atmospheres, potential records of ancient geologic events are lost either due to heavy cratering, as seen on Callisto^6^ and Ceres^7^, or through frequent tectonic resurfacing resulting from tidal dissipation, as seen on Enceladus^8^ and Europa^9^.

The New Horizons flyby of the Pluto system revealed that Charon, Pluto’s moon, is far from saturated crater density due to a decreased influx of impactors in the Kuiper Belt and has an interpreted surface age of ∼4.0 Ga^10,11^. This suggests that the surface of Charon is a promising candidate for recording the early orbital and thermal history in its geologic records. Charon exhibits a topographic dichotomy of rugged northern highlands and smoother southern plains^10,12^. Previous studies proposed that Charon has undergone global extension accompanied by cryovolcanism^10,13,14^. The scarps and troughs were interpreted as normal fault scarps and grabens, and the southern plains were resurfaced by the emplacement of cryolava. Assessment of tidal stress influence on Charon’s surface finds no correlation between observed tensile fracture patterns and those induced by eccentricity tides^15^, while the influence of true polar wander remains to be explored^16^. Besides extensional features, detailed geomorphologic mapping in the northern highlands of Oz Terra recognized arcuate ranges with a half-crater structure^17^. These ranges resemble the lobate scarps on Mercury^18^, which indicate a potential compressive tectonic origin. The predominant north-south orientation and confinement to low latitudes suggest a potential link to despinning-related stresses^17^. However, the geometry, kinematics, and potential dynamic origins of these thrust systems, as well as their potential connection to early tidal evolution, remain unexplored.

In this study, we quantitatively examine the potential of despinning-induced stress to develop compressional tectonic features on Charon and infer Charon's early thermal state and evolution. To apply this framework to Charon, we first characterize the topographic expression of the arcuate ranges and justify using an elastic dislocation model to simulate surface deformation. Second, we infer the geometry and kinematics of subsurface thrust faults from tectonically induced topography, using the elastic dislocation model to constrain both the ice shell thickness and the strain accommodated by faults. Third, we convert the inferred strain into an estimate of the decrease in the equatorial radius, from which we derive the corresponding change in flattening due to rotational evolution. Fourth, we calculate the despinning-induced stress fields based on the inferred flattening, predict the orientations and spatial distribution of tectonic features, and compare these predictions with the mapped tectonics in Oz Terra, Charon. Lastly, we discuss the implications of our results for the early orbital, tectonic, and thermal evolution of Charon, predating the previously proposed phase of global extension^19–21^.

## Results and discussion

### Characteristics of the arcuate ranges

**Fig. 1:**
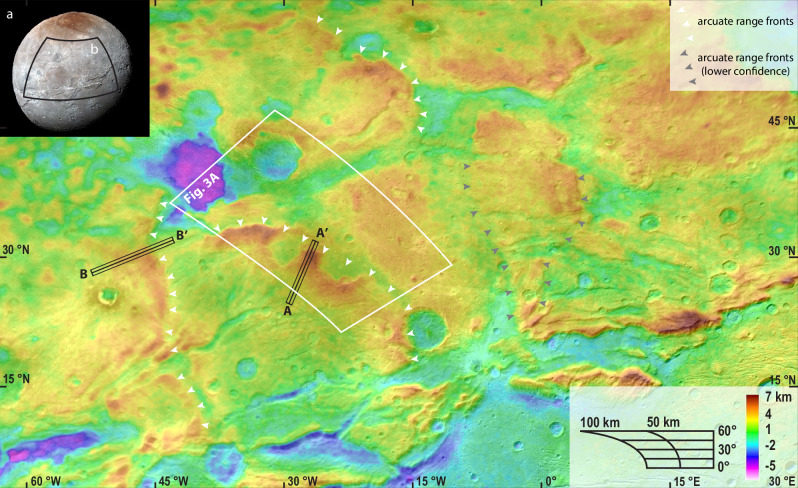
Regional topographic map of the lower-latitude region on Oz Terra, Charon. The background map consists of a color-coded digital elevation model overlaid on an optical image mosaic^24^, showing arcuate ranges and the locations of swath profiles. White arrows indicate the fronts of arcuate ranges (arcuate range *I*, *II*, *III*). Gray arrows indicate arcuate features of lower confidence (arcuate range *IV*, *V*). Arcuate ranges appear as north-northwest trending higher elevation ranges (in brownish color), convex towards east, of lengths around 200 km. A half-crater structure is present along the middle section of the arcuate range *I*. Profiles A-A’ and B-B’ mark the locations of the topographic swaths across the arcuate ranges shown in Fig. 2. Inset (**a**) shows an enhanced color image of Charon’s encountered hemisphere, with the areas of the regional map in (**b**) outlined in black.

We performed a systematic analysis of landforms in the low to mid-latitudes of Oz Terra, Charon’s northern highland, and identified large-scale ( > 10 s km) topographic features that can be divided into two groups (see Methods). The arcuate ranges on Charon appear as positive relief landforms with characteristic morphology, which are different from other large-scale landforms (arcuate ranges I–V in Fig. 1, Methods). In map view, these arcuate ranges show consistent north-northwest trends and are convex toward lower-elevation lands to the east, with lengths over 200 km and widths of ∼ 50 km. The truncation of the arcuate ranges along their southern ends by east-trending troughs or scarps^17^ suggests their earlier development than the extensional fractures and the resurfacing event that formed the Vulcan Planitia, which likely dates back to ∼ 4.0 Ga [e.g. 10].

**Fig. 2:**
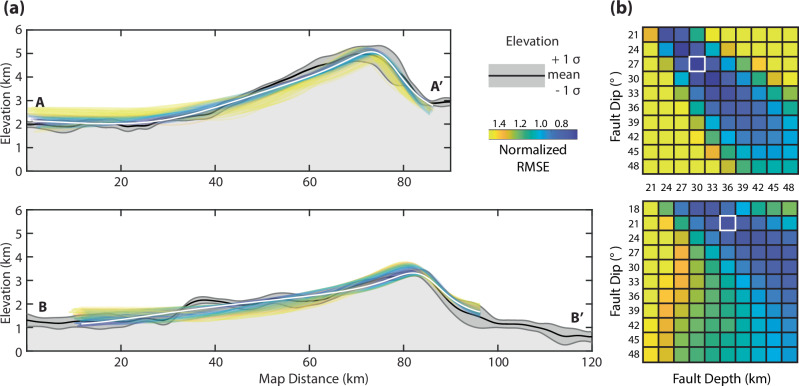
Topographic profiles and model fits across arcuate ranges. **a** Comparison of observed and modeled topographic swath profiles across the two arcuate ranges. **b** Colormap illustrating the goodness-of-fit across a range of fault root depths and fault dip angles. In (**a**), black curves represent the observed mean topographic profiles, and the shaded regions indicate the ± 1 standard deviation (*σ*) along the map swaths. Colored lines show the modeled profiles, with colors corresponding to fit quality in (**b**). Blue indicates better fits, and yellow indicates poorer fits based on normalized root mean squared error (RMSE). The best-fit topographic profile and model parameters are highlighted in white (see Methods for details).

**Fig. 3:**
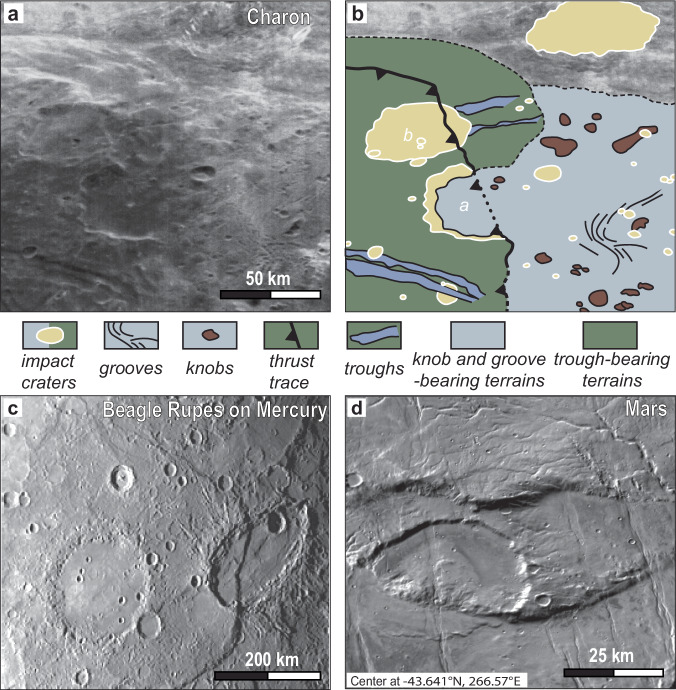
Comparison of half-crater structure on Charon with planetary analogs. **a** The half-crater landform along the central arcuate range shown in the highest-resolution LORRI image. **b** Geomorphological map with two modified craters (*a* and *b*) from the landforms shown in (**a**). Only the western half of the crater remains discernible, with the eastern half missing. The western half of the crater is located in the trough-bearing terrains, while the eastern half is probably buried under the plain materials (knob and groove-bearing terrains). A vertical offset surpassing the crater depth is required for such contrast, which can be generated by thrusting. Crater *b* is a flat-floored crater with a scarp cut across the crater floor, with the left side slightly uplifted. **c** Image showing the Beagle Rupes on Mercury, where a lobate scarp cuts across a crater. Interpreted thrust that produced the lobate scarp landform also elevated the left half of the crater relative to the right half^45^. **d** Half crater along the margin of the Thaumasia Highlands on Mars, with the northern half elevated and preserved, while the southern part is missing, attributed to a compressional tectonic origin^46^. **a** is from PIA10939 in NASA’s Photojournal database^17^.

We extracted topographic swath profiles across two arcuate ranges and analyzed their topographic characteristics (Fig. 2, locations of the profiles A-A’ and B-B’ are shown in Fig. 1). The profiles consistently exhibit their gently sloping back limbs ( < 5^◦^) and steeper forelimbs ( < 30^◦^), reminiscent of fault propagation folds on Earth where surface folding accommodates the compressional strain of underlying blind thrusts^22^. These morphological characteristics of the arcuate ranges are similar to the lobate scarps on Mercury [e.g. 18, Fig. 3c], whose origin is also attributed to compressional tectonics rather than extensional tectonics (Methods). The asymmetry indicates east-verging thrusts underlying these arcuate ranges. However, arcuate ranges on Charon differ from these planetary analogs in their larger scales and less along-strike variations. The typical spans of the back limbs and forelimbs are ∼ 60 km and ∼ 15 km, respectively, while the topographic relief reaches 2 km (Fig. 2). The larger scale indicates deeper-rooted thrusts and probably larger shortening amounts, similar to the thick-skinned thrusts on Earth, such as the Wind River Thrust in Wyoming, where basement rock gets involved in the fault propagation folds^23^.

### Modified craters along the arcuate ranges

Additional supporting evidence for the compressional origins of the arcuate ranges comes from the modified craters along arcuate range I (Fig. 1, 3a, b^17^,). Two craters with diameters around 50 km exhibit post-cratering modifications (crater a and b in Fig. 3b). The most prominent landform along arcuate range I is the half-crater structure located in the middle of the range (crater a in Fig. 3b). The western half of the crater is well-preserved and seemingly undeformed, while the eastern half is missing. The surface of the region east of this half crater is distinct from other rugged highlands in its smoother surface texture, which was interpreted to have been resurfaced similar to Vulcan Planitia^10,24^. The eastern half of the crater could have been mantled by resurfacing materials or degraded into significantly warmer or weaker materials, but it still requires a vertical offset greater than the crater depth or a sharp boundary to achieve such a distinct contrast. Thrusting can generate vertical offset without significantly deforming the crater morphology, as seen along the Bagel Rupes on Mercury (Fig. 3c) and within Thaumasia Highland on Mars (Fig. 3d). Though east-dipping normal faulting is capable of generating the observed vertical offset, it is a less likely mechanism, as the anticipated normal fault scarps are not observed, while the range front is arcuate instead of the anticipated polygonal geometry in the proposed intact primordial crustal blocks model^13^.

Crater b in Fig. 3b appears as a flat-floor crater, which locally obliterates the positive topography generated by the arcuate range. However, a scarp cut across the crater floor, which connects the arcuate range fronts to its north and south (Fig. 3a), may represent the surface traces where the thrust ruptures to the surface. Moreover, the west-side-up motion along the scarp is consistent with the interpreted east-verging geometry of the thrust. The contrast between the two craters suggests their relative ages, with the half-crater a predating the arcuate range formation, while the flat-floored crater b is syntectonic. Both craters show limited relaxation (Fig. 1), suggesting non-severe post-thrusting viscous relaxation along the arcuate ranges. A similarly sized crater at the southern end of arcuate range I overprints its topography (Fig. 1) and therefore appears to postdate the development of the range.

### Subsurface structure inferred from surface topography

We inferred the geometry and kinematics of the subsurface structures by fitting the topographic profiles across the arcuate ranges modeled from an elastic dislocation model^25,26^. An elastic model is assumed for investigation based on surface relaxation that is not severe, as well as the literature standard of utilizing an elastic baseline in the absence of pronounced or obvious viscous requirements. We adopt a similar approach to fit the surface deformation caused by underlying thrusts, as recorded in elevated river terraces on Earth [e.g., ref. ^27^] and lobate scarps on Mercury^28^.

We first extract two topographic profiles across the arcuate ranges that preserve pristine morphology, are unaffected by later cratering, and occur in areas with relatively high topographic resolution (profiles A-A’ and B-B’ in Fig. 1, see Methods: Topographic Analysis and Identification of Candidate Arcuate Ranges). We then compare these profiles with modeled surface deformation. Using an elastic dislocation model that incorporates ice rheology, we assess the displacement from topographic relief across the profiles and simulate topographic profiles by varying two vital parameters: fault dip and fault root depth (Methods^28^,). Then we assess the quality of the fits by calculating the normalized root-mean-square error. Along arcuate range I (profile A-A’), the fit achieves a normalized RMSE value down to 0.8 with the fault dip angles of 24^◦^ − 30^◦^ and the fault root depths of 27–33 km, with the best fit representing a fault dip of 27^◦^ and a fault root depth of 30 km. Along arcuate range II (profile B-B’), the fits capture most of the topographic trends, except for undulations along the back limb (Fig. 2). The best fit for profile B-B’ represents a fault dip of 21^◦^ and a fault root depth of 36 km. The choice of model variables, procedures, and associated uncertainties are described in the Methods.

### Despinning origin for the thrusts

**Fig. 4:**
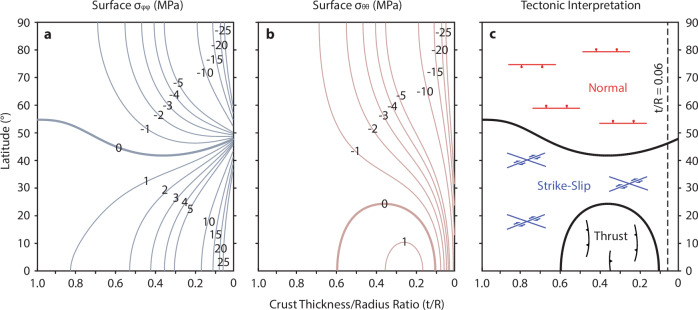
Despinning-induced surface stresses as a function of crustal thickness and latitude for Charon. **a** Stress in the east-west direction (*σ*_*ϕϕ*_) and **b** stress in the north-south direction (*σ*_*θθ*_) were calculated for a uniform, spherical icy Charon. The contour lines map out the stress in MPa for the given ratio of crustal thickness (t) to the mean radius (R). Compression is positive. **c** Tectonic interpretation based on the calculated stresses. Equatorial thrust faults with north-south strikes are predicted for t/R between ∼ 0.1 and ∼ 0.6. The Strike-slip tectonic province encircles the lower-latitude thrust tectonic province and extends to ∼ 50^◦^ in latitude. Normal faults with east-west strikes are predicted to dominate the polar region. Charon’s estimated minimum crustal thickness corresponds to a ratio of ∼ 0.06 (vertical dashed line), predicting lower-latitude strike-slip faults and polar normal faults.

We calculated surface stress in both east-west (*σ*_*ϕϕ*_; Fig. 4a north–south (*σ*_*θθ*_; Fig. 4b) from despinning as a function of crustal thickness and latitude following Melosh’s despinning tectonic model^1^. We utilized the change in Charon’s spinning state based on our estimated subsurface structure and horizontal strain. The estimated ∼ 30 km fault root depth serves as the lower bound for the elastic crustal thickness, and the initial flattening of ∼ 0.04 is derived from the strain accommodated by the thrusts (see Methods).

**Fig. 5:**
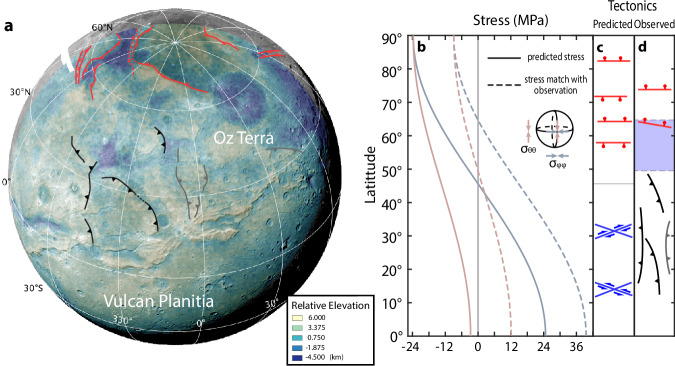
Global tectonic patterns from despinning-induced stresses on Charon. **a** Major tectonic features observed in the northern highlands of Charon, including polar region scarps (red), lower-latitude arcuate ranges (black), and potential lobate scarps (gray). Figure modified from Fig. 2 in ref. ^13^, which is licensed under CC BY 4.0. **b–d** Latitudinal variations of principal stresses and comparison between observed and predicted tectonic patterns. In (**b**), despinning-induced principal stresses: *σ*_*ϕϕ*_ denotes the stress in the east-west direction, *σ*_*θθ*_ denotes the stress in the north-south direction. The third principal stress *σ*_*rr*_ in the radial direction is 0 at the surface (vertical gray line). **c** The predicted tectonic pattern is based on the relative magnitudes of the calculated principal stresses, including lower-latitude strikeslip regimes, and polar extension. **d** The observed tectonic pattern extracted from the encountered hemisphere shows lower-latitude thrusts and polar extensional features. An additional compressive stress component is needed to match the observed extent of thrusts on Charon (dashed curves in b), which may be attributed to global contraction. Discrepancy remains in the mid-latitude region (shaded section in d), where the anticipated strike-slip dominant tectonics is not observed.

Based on the relative magnitude of the despinning-induced principal stresses^1^, we can predict the spatial extent and types of tectonic provinces (Fig. 4c). Despinning induces a more compressive *σ*_*ϕϕ*_ than *σ*_*θθ*_ within the elastic shell at all latitudes, which generally predicts an equatorial thrust-tectonic province, a lower-latitude strike-slip dominant province and a higher-latitude extensional province. For Charon, an equatorial Thrust-tectonic province exists for crustal thickness between 0.1 and 0.6 of Charon’s mean radius (Fig. 4). The observed arcuate ranges show consistent north-northwest trends and are confined to the lower-latitude region *<*∼ 45^◦^N as shown in Fig. 5a, which aligns with the predicted equatorial compressional tectonics of a despun planet^1^.

The despinning origin of tectonic features is further supported by the landforms observed in the north pole region of Charon. The scarps of the polar basin appear semi-circular (red-colored scarps in Fig. 5a), which aligns with the predicted east-west trending normal faults in the polar region. Higher-latitude limb images show significant topographic relief along scarps^13^ (Fig. 5a), which also have a roughly east-west trend, consistent with the predicted pattern.

However, the northernmost arcuate ranges on Charon extend to ∼ 50^◦^N (Fig. 5d), reaching higher latitudes than the predicted ∼ 25^◦^N northernmost extent (Fig. 4c). In addition, an elastic thickness of 36 km for Charon’s icy shell corresponds to a crustal thickness(t)/radius(R) ratio of ∼ 0.06 (Fig. 4c). With this ratio, the stress field (Fig. 4c and Fig. 5b) indicates a strike-slip dominant region between the equator and ∼ 45^◦^N latitude, and an extensional tectonic region ranging from ∼ 45^◦^N to the pole with east-west trends (Fig. 5c).

One plausible explanation for these differences is that the despinning process may be accompanied by global contraction^2^, which can add additional compressive stresses at all latitudes. To match the observed extent of the thrust tectonics province, an additional ∼ 12 MPa compressional stress (*σ*) is required (dashed lines in Fig. 5b). A reduction of ∼ 0.55 km in radius, corresponding to a compressional strain (*ϵ*) of ∼ 0.09%, can produce this magnitude from the relationship *σ* = *E/*(1 − *ν*) × *ϵ* (*E* is Young’s modulus, *ν* is Poisson’s ratio). This strain is about an order of magnitude lower than the proposed ∼ 1% areal strain of later global extension on Charon^13^ and is compatible with the proposed thermal evolution^20^. Further analysis of uncertainties in the fitted parameters and estimated crustal thickness, together with their influence on the required compressional stress, is provided in the Methods.

Another discrepancy between the observed and predicted tectonic patterns is the absence of the mid-latitude strike-slip system in Fig. 5. We suspect that the strike-slip systems may have developed in the mid-latitude region of Oz Terra. However, they are indiscernible either because they produce limited vertical displacements across strike-slip faults or because surface ruptures have been obscured by subsequent space weathering. Alternatively, the strike-slip systems may have been incorporated into thrust systems, with the arcuate ranges formed by transpressional faulting^29^.

### Timing of thrust faulting and implications for the early thermal state of Charon

The despinning-induced tectonics is anticipated to be one of the ‘earliest’ tectonic events following Charon’s accretion. The despinning process of Charon was inferred to have reached its present stage relatively quickly, based on orbital dynamic studies, on a timescale of 1 to 10 Myr^21,30^. The preservation of tectonic features induced by despinning agrees with the surface age of ∼4.0 Ga for Charon inferred from the crater density^10,11^. The truncation of the arcuate ranges by the scarps and grabens along the dichotomous boundary, along with the absence of arcuate ranges in the geologically younger southern plain, suggests that despinning tectonics ceased before the proposed global extension and cryovolcanism^10,14^.

The despinning model helps to explain the preferred east-west trending of the scarps and grabens related to global extension, which remains to be addressed in the global extension model^13^. This configuration may have been controlled by residual stress from despinning, which served as a background stress field during global extension. For despinning-induced stress, the stress component in the east-to-west direction is always more compressive or less extensive than that in the north-to-south direction (Fig. 5B). The integration of the residual stress from despinning and the uniform tensile stress caused by global extension will result in the lowest stress in the north-south direction, which will preferentially form extensional features in the east-west direction. In addition, the alignment of latitudinally varying tectonic features to the despinning-induced tectonic pattern indicates limited true polar wander^16^ of Charon after its despinning process. The larger compressional strain along arcuate range I, closer to the sub-Pluto point, may have partially resulted from orbital recession^31^.

The tectonics in Oz Terra record both the early thermal state and spinning state of Charon. The best estimate of fault root depth constrains the minimum elastic thickness of Charon’s ice crust shell to be around ∼30–36 km, an order of magnitude greater than that estimated from flexure^13^. The ~2 km estimate from flexural fitting may have captured the thermal anomaly caused by cryovulcanism during global extension. The around-30 km estimate implies a thick, rigid ice shell for Charon during the early stage, which is consistent with the limited degree of crater relaxation. Such a thick ice shell favors a cold initial thermal state (“cold start”), as predicted by the thermal evolution models^19,20,32^. This scenario is compatible with the giant impact hypothesis for Charon’s origin proposed by refs. ^21,33^, in which a giant impact produced a thermal anomaly primarily on Pluto and resulted in the accretion of Charon from relatively cold ejected material. A cold start is further congruent with the need for global contraction to accompany despinning in reproducing the observed tectonic patterns. The inferred global contraction could have resulted from porosity reduction or from the formation of a subsurface ocean during the cold start phase^20^.

The strain accommodated by the thrusts suggests an initial flattening of ∼0.04, which corresponds to an initial rotation period of ∼14.25 h for Charon. This rotation period estimate is comparable to the 12 h and 14 h rotation periods estimated for Arrokoth^34^. Putative results from the kiss-and-captures model for Charon-forming impact simulations^33^ also show compatible estimates, where the rotation period of Charon varies around 10 h immediately after the Charon-forming giant impact.

Our work presents an approach for quantifying despinning-induced strain and stress on planetary bodies by adapting structural geology techniques developed for terrestrial settings^26,35^. The distribution of tectonic provinces on Charon suggests that despinning was accompanied by global contraction, supporting a cold start for Charon. Although our work provides a plausible explanation for the tectonic patterns preserved in Charon’s northern highlands, further studies are needed for a more comprehensive understanding of the thermal-mechanical evolution of the crust. In particular, our criteria for identifying tectonic patterns follow Anderson’s theory instead of incorporating a more realistic brittle failure mechanism, such as the Griffith criterion^36^. Future work could integrate failure envelopes or stress-dependent rheological models to better capture the mechanical behavior of the ice shell and improve predictions of fault style and distribution on Charon.

## Methods

### Data sources

The data used in this project are based on the combined global image mosaic and digital elevation models (DEMs); both of which are products of ref. ^24^. The resolutions of the global image mosaic vary from ∼154 m/pixel to ∼1460 m/pixel. Meanwhile, the DEMs used in this study have a uniform map-view resolution of 300 m/pixel, while the vertical precisions vary between 100 m/pixel and ∼1400 m/pixel^24,37^.

As the DEM product was constructed using stereophotogrammetry based on optical images, it has a lower resolution. We overlay the global image mosaic on top of the color-coded DEM in ArcGIS to integrate the information from these two products. The mapping area spans from 8^◦^N to 45^◦^N, from 295^◦^E to 355^◦^E, following the coordinate system defined by ref. ^24^. The vertical precision of the DEM in this area varies from 100 m/pixel in the lower right corner to 875 m/pixel along the left edge. Detailed processing and data product information are provided by ref. ^24^.

### Topographic analysis and identification of candidate arcuate ranges

We examined the topography of the entire Oz Terra region and comprehensively identified large-scale linear features by overlaying the digital elevation model (DEM) on a DEM-derived slope map (Fig. S1). We consider linear features with length greater than 50 km, where elevation change matches with >15° slopes. These features can be generally divided into two types: (type 1) steep slopes ( > 35°), large elevation change ( > 3 km), and short-length segments ( ~ 50 km); (type 2) moderate slopes ( < 35°), small elevation change ( < 3 km), and long lengths ( > 200 km). Previous mapping works^13,37^ captures all the type 1 features (e.g., feature a–d in Fig. S1), but missing several of type 2 features (e.g., feature I, IV, V in Fig. S1).

Based on the morphology of sharp crest lines adjacent to steep planer slopes, *type 1* features were interpreted as normal fault scarps^10,13,37^, which is further supported by the observation of extensive graben-like troughs. The moderate slopes and missing sharp crest lines of type 2 features seem to contradict an interpretation of normal fault scarps. Instead, their correlation with ranges and arcuate traces resembles the topography of thrust-origin landforms. The contrasting morphologic criteria used to distinguish these two landform groups are summarized in Table S1.

Examples of Type 1 include linear or lobate scarp features that can be readily identified in the slope map (a–d in Fig. S1). Group *a* is a series of tectonic features trending northeast, often referred to as the tectonic belt^10^. These features are characterized by steep scarps ( > 35°) and often host sharp crests (Fig. 1), which are typical of normal-fault-related landforms. Their planar slopes and concave-toward-lowland morphology further support this interpretation. Group *b* consists of a series of northeast-trending broad troughs in the mid-latitude region. The traces marking the elevation change are relatively straight rather than arcuate in shape and often occur in pairs, bounding the trough. Although neither distinct planar scarps nor sharp crest ridge lines are developed, an arcuate range interpretation is less likely because thrusts rarely develop in paired configuration opposing one another. Group *c* comprises a set of sub-parallel northwest-trending scarps adjacent to the tectonic belt. These features show linear or southward-concave sharp crest lines consistent with a normal-fault origin. Feature *d* appears as an east-trending arcuate front with a rounded range top. This feature, however, is connected to a concave, east-trending planar scarp, with a steeper slope that bounds part of Group b. Thus, feature d is considered a less likely candidate for an arcuate range.

We identified Type 2 features based on their morphologic characteristics (Table S1) and selected them as potential thrust-induced arcuate ranges. Arcuate ranges I and II exhibit gentle frontal slopes that are convex toward the adjacent lowlands and have rounded range tops. Arcuate range III shows similar characteristics but has steeper frontal slopes. Arcuate range IV and V also appear as convex features, marking the abrupt termination of other landform features. However, the morphologic patterns along these two traces are less pronounced than along the others. Arcuate range I and II are selected for topographic analysis, and we extracted two topographic profiles (A-A’ and B-B’ in Fig. 1). We selected range I and II because they are located within relatively higher-resolution DEM regions (e.g., ~ 400 m)^24^. They have pristine, characteristic asymmetric topographic profiles unaffected by post-cratering, and a similar reference elevation in the foreland and hinterland. We did not include arcuate range III due to a low reference elevation in its foreland, potentially due to the juxtaposition with extensional features of group b. The locations of arcuate ranges I, II, and IV are considered to estimate the spacing between arcuate ranges for shortening calculation (see details in Methods: Despinning-induced Stress Field Calculation).

### Elastic dislocation model

We use an elastic dislocation model to simulate the north-south trending thrusts observed in the lower-latitude region (arcuate range I, II in Fig. 1). These arcuate ranges have a steeper forelimb and a gently sloping back limb in cross-section view. This type of geometry is reminiscent of the fault propagation fold model^22^, where slip along an underlying blind thrust results in folding in the hanging wall. The steeper forelimb indicates the vergence direction of the underlying thrust, while the slope of the back limb roughly represents the dip of the thrust. We examine a range of potential underlying fault geometries and kinematics and assess whether the model can reproduce the observed topography. Based on the fault geometry and kinematics, we estimate the shortening accommodated by these thrusts.

We employ the elastic dislocation model proposed by Okada^26^ (hereafter, the Okada model). The analytical solutions derived by Okada^26^ express the displacement field at certain points in the elastic medium as a function of the fault parameters and the elastic constants. To apply this model, we assume that: (1) the physical properties of the medium are constant with depth. (2) The deformation is accumulated with a series of seismic events that have integrated the slip along the fault. (3) Viscous relaxation is negligible. Based on these assumptions, the deformation can be simplified to a single time-independent event.

We utilize the open-access elastic dislocation program COULOMB 3.4 available at USGS^25,38^. For the model setup, we assume Young’s modulus *E* to be 9 GPa, as measured through Brillouin spectroscopy^39^, and set the Poisson’s ratio at 0.33. The main input components of fault parameters include (1) fault dip, (2) slip magnitude along the fault, (3) slip distribution along the fault, (4) fault tip depth, and (5) fault root depth. With this input of the fault geometry and slip, the model generates a corresponding displacement field within the medium, from which the surface displacement can be extracted. As discussed in ref. ^28^, besides the slip amount that can be estimated from topographic relief, fault dip and fault root depth are the two major parameters that control the configuration of the displacement field.

We explore the parameter space of fault dip and fault root depth to fit the observed uplifted topography across the arcuate ranges. Topographic profiles are extracted from the DEM as 10-km-wide swath profiles using the SwathObj function in MATLAB TopoToolbox^40^. For each position along the profile, we calculate the mean elevation and a 1-standard-deviation (1*σ*) range to account for topographic variations within the swath width. The mean elevation within the swath width serves as the reference profile for model fitting. The average of the one-standard-deviation values along the profile (1*σ*_*p*_) represents the mean range of observed topographic variability across the swath width. The model misfit is quantified as the root-mean-square error (RMSE) between the measured and modeled elevations along the profile. We then compute the normalized root mean square error (nRMSE) as the RMSE divided by 1*σ*_*p*_ from the profile (*nRMSE* = *RMSE/*(1*σ*_*p*_)). Model quality is evaluated using the normalized RMSE for each profile, with the best fit corresponding to the lowest value. A normalized RMSE < 1 indicates that model uncertainties fall within the range of observed topographic variability and allows direct comparison of fit quality among different profiles.

In the fitting process, we focus on the major fault, while the effects of the minor antithetic faults are not explicitly modeled. The topographic undulations along the backlimb in profile B-B’ may have been induced by the antithetic thrusts (see the 30–50 km section in B-B’ profile in Fig. 2). The presence of antithetic faults in profile B-B’ likely results in systematically higher RMSE values and broader ranges of acceptable model parameters compared to those in profile A-A’.

### Despinning-induced stress field calculation

We adopt the calculation of despinning-induced stresses, following the approach of Melosh’s despinning tectonic model^1^. As a planet despins, the decrease in its flattening results in the relaxation of an equatorial bulge. Such deformation generates differential stresses in the elastic shell of the planetary body that may reach the strength of the shell materials. For detailed assumptions and derivations, see ref. ^1^.

Here, we outline the modifications we made to apply this method to Charon. We first relate the change in planetary flattening to the change in spinning state. Then, we infer the initial spinning state based on the estimated compressive strain accommodated by the equatorial thrusts. Lastly, we calculate the despinning-induced stress field based on the elastic ice shell thickness, the change in spinning state, and ice properties. The detailed descriptions of each step are provided below.

We first calculate present-day flattening based on the current rotation rate of Charon. For a uniform-density body, the rotation rate can be related to the flattening^41^ as:

1 $$f=\frac{{R}_{e}-{R}_{P}}{R}=\frac{5}{4}\frac{{\omega }^{2}{R}^{3}}{{GM}}$$

where *f* is the flattening of the planetary body, *R* represents the mean radius of the planetary body, *R*_*e*_ is the equatorial radius, *R*_*p*_ is the polar radius, *ω* is the rotational speed of the body (angular velocity), *G* is the universal gravitational constant, and *M* is the total mass of the planetary body. Rearranging the equation, we can derive:

2 $$\omega={\left(\frac{4{fGM}}{5{R}^{3}}\right)}^{\frac{1}{2}}$$

The rotational period *T* can be expressed in terms of the angular velocity *ω* as follows:

3 $$T=\frac{2\pi }{\omega }=2\pi {\left(\frac{5{R}^{3}}{4{fGM}}\right)}^{\frac{1}{2}}$$

At present, Charon is tidally locked with Pluto, with a rotational period of ∼153.3 h. Assuming a hydrostatic state, the corresponding present-day flattening *f*^′^ is 3.563× 10 − 4.

Second, we estimate the initial flattening *f* from the initial equatorial radius of the planetary body, which is derived from the pre-strain state. On Charon, the strain accommodated along the arcuate ranges can be estimated using the balanced cross-section method [ref. ^35^, also Fig. S2]. The area of positive topography in cross-section should equal the horizontal shortening amount in reference to a detachment depth. Here, we assume the fault root depth as the detachment depth and derive a horizontal shortening of ∼ 2.9 km accommodated by the thrust. Taking the average spacing of ∼ 280 km between arcuate ranges I, II and IV in Fig. 1 as the reference length, this shortening amount corresponds to a regional compressional strain of ∼1%. Assuming such a strain is uniform across the equatorial region of Charon, a change in equatorial radius ∼1% will be required. The increase in equatorial radius will result in an estimated initial flattening *f* of 4.075 × 10^−2^. This flattening corresponds to an initial rotation period of ∼ 14.25 h.

Lastly, we calculate the despinning-induced stress based on the derived elastic ice shell thickness and change in flattening. We assume that Charon had an elastic ice shell over a subsurface ocean or a viscous ice layer after its formation. Thus, we utilized the ‘thick lithosphere scenario’ of the despinning tectonic model^1^, which relates the change in the flattening to the change in the spinning state of a planetary body with crustal thickness at a certain ratio to the mean radius of the planet. We calculate lithospheric despinning stresses following the appendix of^1^. As discussed in previous studies^1,3^, density difference between the crust and mantle plays a negligible role in changing the despinning-induced stress field. We simplified the model to consider a uniform-density Charon with density of ∼ 1700 *kg/m*^3^^12^. We adopt a Young’s modulus of 9 GPa^39^ and a Poisson’s ratio of ∼ 0.33^42^, with a corresponding shear modulus of ∼ 3.38 GPa (see Table 1). The calculated stress as a function of latitude and crustal thickness (as the ratio to the radius; t/R) is presented in Fig. 4. A 36 km- thick elastic crust represents a ratio of 0.06, resulting in the stress shown in Fig. 5.

**Table 1:** List of parameters and constants used in the models

Properties	Symbol	Value	[units]
Flattening	*f*		
Change in flattening (with crust)	∆*f*_*c*_		
Mean Radius	*R*	606 ± 1^12^	[km]
Equatorial radius	*R*_*e*_		[km]
Polar radius	*R*_*p*_		[km]
Mean density	*ρ*	1707 ± 17^12^	[kg m^−3^]
Mass of Charon	*M*	1.5857 × 10^21^^44^	[kg]
Gravitational constant	*G*	6.67 × 10^−11^	[N kg^−2^ m^2^]
Rotational speed	*ω*		[rad s^−1^]
Rotational period	*T*		[s]
Young’s modulus	*E*	9^39^	[GPa]
Poisson’s ratio	*ν*	0.33^42^	
Shear modulus	*µ*	3.3835	[GPa]
Ice shell thickness	*t*	36 (this study)	[km]

### Uncertainties in elastic modeling and inferred stress estimates

We acknowledge that uncertainties exist in our elastic modeling approach used to estimate crustal thickness, which lead to subsequent uncertainties in the inferred despinning-induced stresses and the additional stress required. In this section, we explain the sources of these model estimate uncertainties, justify our modeling choices and the adopted model setup and parameter ranges, and discuss their implications for our conclusions.

First, we use the best-fit values of the elastic thickness of Charon’s crust from an elastic dislocation model applied to the topographic features of Charon. However, there is uncertainty in the estimate of elastic thickness due to the trade-off between model parameters. For example, fitting topographic profiles via the Okada Model shows trade-offs between fault dip angles and fault root depth (Fig. 2), where the influence from increased fault dip can be compensated by the increase of fault root depth. The nRMSE values indicate a trend of higher values (worse fits) as fault parameters continue to increase after fault root depth surpassing ~40 km and fault dip surpassing ~35°. Varying the fault root depth by ~ ±5 km around the chosen best fit value of 36 km corresponds to ~ ±0.01 deviation in crust thickness/radius (t/R) ratio. This range results in relatively small latitudinal variation extent of tectonic provinces (less than ±1° in Fig. 4). The uncertainties from the trade-offs between parameters do not cause significant deviation from the best-fit pair case that we presented in Fig. 5.

Second, we assumed the fault root depth as a lower bound for the elastic thickness of the icy crust. However, mechanical stratification may localize detachment along weak layers, allowing the elastic thickness to differ from the inferred fault depth. To explore the impact of potential ranges of elastic thickness of Charon’s icy crust, we quantify the influence of varying elastic thickness on the extent of predicted tectonic provinces (Fig. 4) and on the minimum additional compressional stress required to match with the observed tectonic configuration (Fig. S3c). Based on previous studies of Charon’s interior structure [e.g., refs. ^13,19,20^], we examine elastic thickness values ranging from ~ 6 to 240 km, corresponding to t/R values of 0.01 to 0.4. The lower bound ( ~ 6 km) is derived from elastic thickness estimates obtained from flexural fits in a previous study^13^, and the upper bound ( ~ 240 km) corresponds to the inferred total thickness of Charon’s icy shell from interior structure models^20^.

To illustrate the differences, we present two examples with elastic thicknesses (t/R values) of 0.1 and 0.2. For t/R = 0.2, the model predicts compressional tectonic province at equatorial area, strike-slip dominant province in the mid-latitude region, and extensional tectonics around the poles (Fig. S3a). An additional 6 MPa compressional stress is needed to match the stress field with the observed extent of tectonic features. For t/R = 0.1, no equatorial compressional tectonic province is predicted, where an additional 8 MPa compressional stress is needed (Fig. S3b). The required additional stress within possible Charon crust thickness values increases as elastic crustal thickness decreases (Fig. S3c), from ~2 MPa of a 240-km crust to ~26 MPa of a 6-km crust. Although we did not model porosity reduction over the relevant timescale, these estimates are plausible given global contraction due to subsurface ocean growth. Previous studies have estimated ~ 100 MPa accumulated extensional stress from the subsequent refreezing of the subsurface global ocean that induced global extension^43^. Lastly, there is also uncertainty in determining the latitudinal extent of tectonic features. We present their variations (45–49°) shown by the colored curves in Figure. S3c, which shows small ( < 1.5 MPa) variations in the estimation of required stress.

Despite these uncertainties, the range of elastic thickness and required stress values explored in this study remains consistent with plausible magnitudes of global contraction on Charon. Our results, therefore, provide a robust first-order constraint on despinning-induced tectonics and suggest that such signals may be preserved in Charon’s ancient global topography. Future work incorporating porosity evolution and thermal models will further refine these estimates and improve our understanding of Charon’s tectonic evolution.

## Supplementary information

Supplementary Information

Transparent Peer Review file

## Data Availability

The DEM data used in this study is from^24^, which are available at Astropedia - Charon New Horizons LORRI MVIC Global DEM 300m. Optical images are accessible through the NASA Planetary Data System (https://pds.nasa.gov/).

## Code availability

Topotoolbox 2.0 is available at https://github.com/wschwanghart/topotoolbox. The code for Okada model is incorporated in the Coulomb 3 software, available at https://temblor.net/coulomb/.
