# Supplementary material for: Early tidal despinning history recorded in the tectonics of Oz Terra, Charon
Source: Nat Commun. 2026 Jul 14;17:5978. doi: 10.1038/s41467-026-75069-7 (PMC13369154; doi:10.1038/s41467-026-75069-7)
Supplement: Supplementary file 1 — Supplementary Information [file 41467_2026_75069_MOESM1_ESM.pdf]

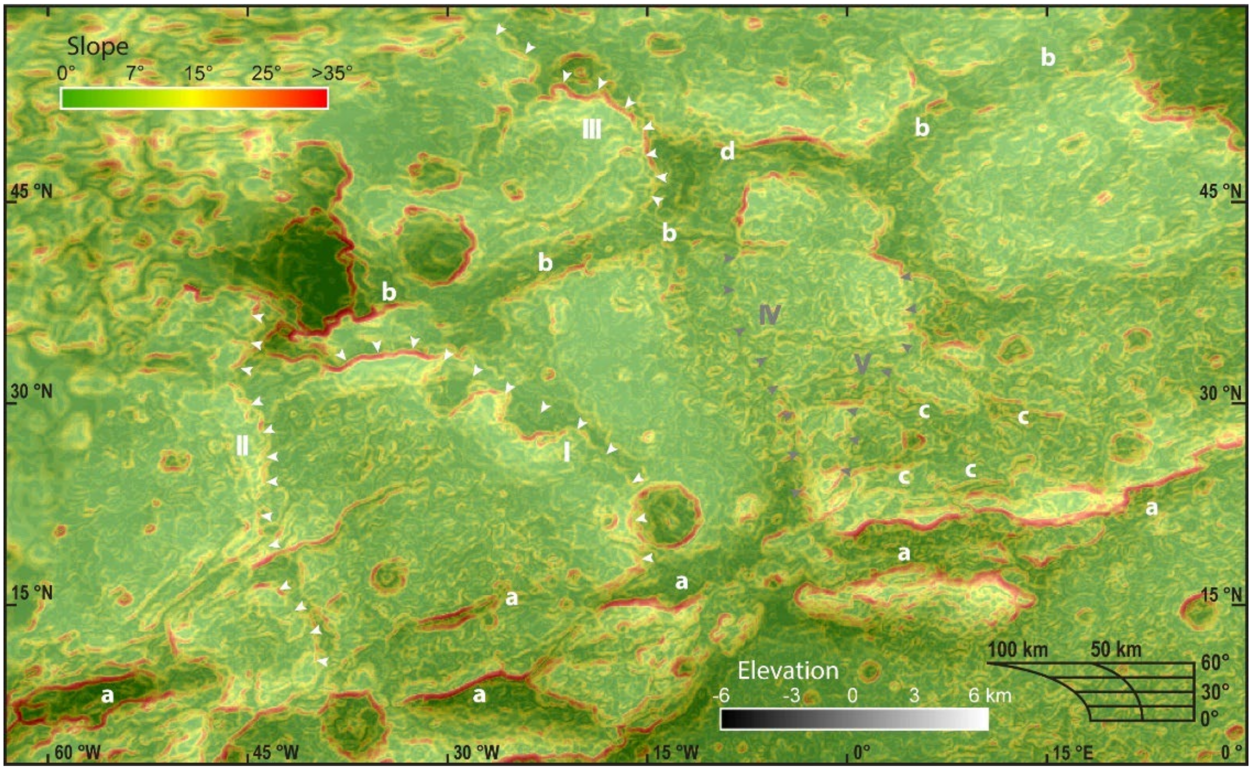

Fig. S1 Slope map of the study area. The map shows the color-coded slope value overlying the digital elevation model of the study area. The slope values were generated based on the Charon Global DEM [1,2] using ArcMap. *Type 1* features are labeled in (*a~d*), which are more likely extension-related features. The white and grey arrows trace out *Type 2* features (*I~V*), same as the potential arcuate range features presented in Fig. 1.

Table S1 Geomorphologic characteristics of arcuate ranges and extensional features

**Landforms**	***Type 1*** **Scarps**	***Type 2*** **Arcuate ranges**
**Topographic lengths**		
- **Along-strike**	- ~50 km	- >200 km
- **Cross -strike**	- ~10 km	- ~30 – 50 km
**Map view traces**	straighter and in segments	acuate
**Topographic relief**	>3 km	<3 km
**Range front slopes**	>35°	<35°
**Slope morphology**	concave upward toward lower elevations	convex upward toward lower elevations
**Ridge crest morphology**	sharp ridge crest lines	broad, rounded ridge tops
**Interpreted fault types**	normal faults	thrust faults

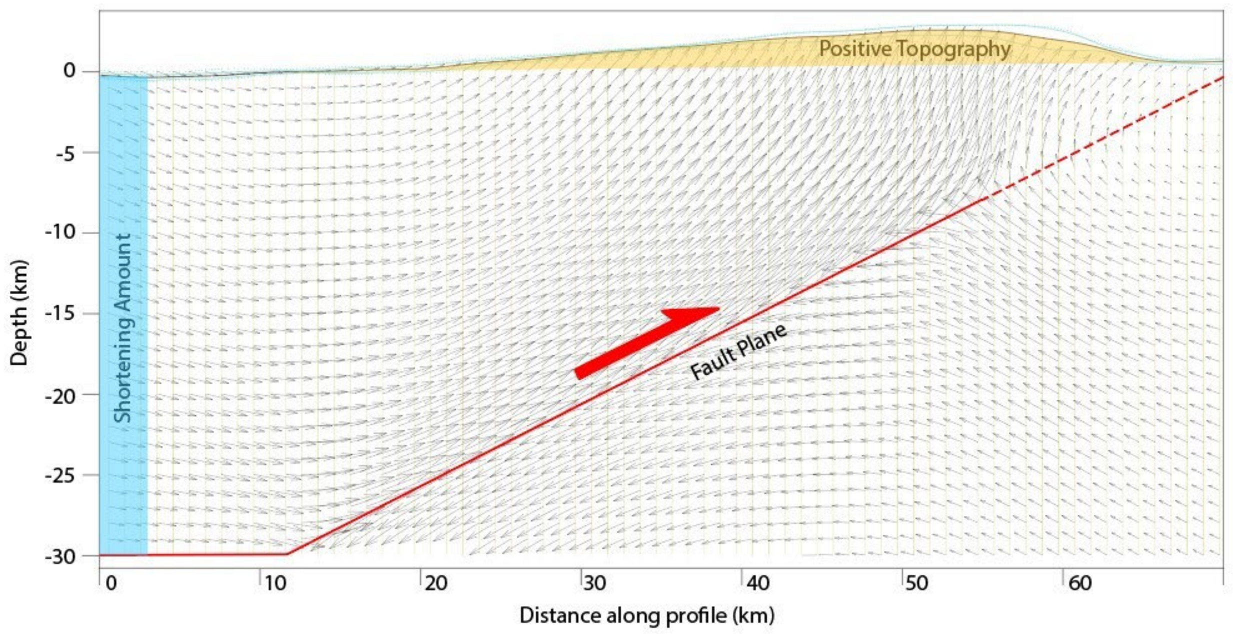

Fig. S2 Illustration on calculating shortening via the balanced cross-section method. The black curve shows the observed topographic mean of swath profile A-A', with blue curves denoting the one standard deviation of topographic variation. The arrows represent the displacement field around the fault. The fault geometry and the displacement field enable the application of the balanced cross-section method to calculate the shortening amount accommodated by the thrust. The area of the positive topography (yellow) is equal to the shortening area in the horizontal direction (blue). Given the fault root depth of 30 km, the regional shortening amount turns out to be ∼ 2.9 km. Referencing the ∼ 280 km spacing of the two analyzed arcuate ranges in Fig. 1, the corresponding regional strain is ∼1%.

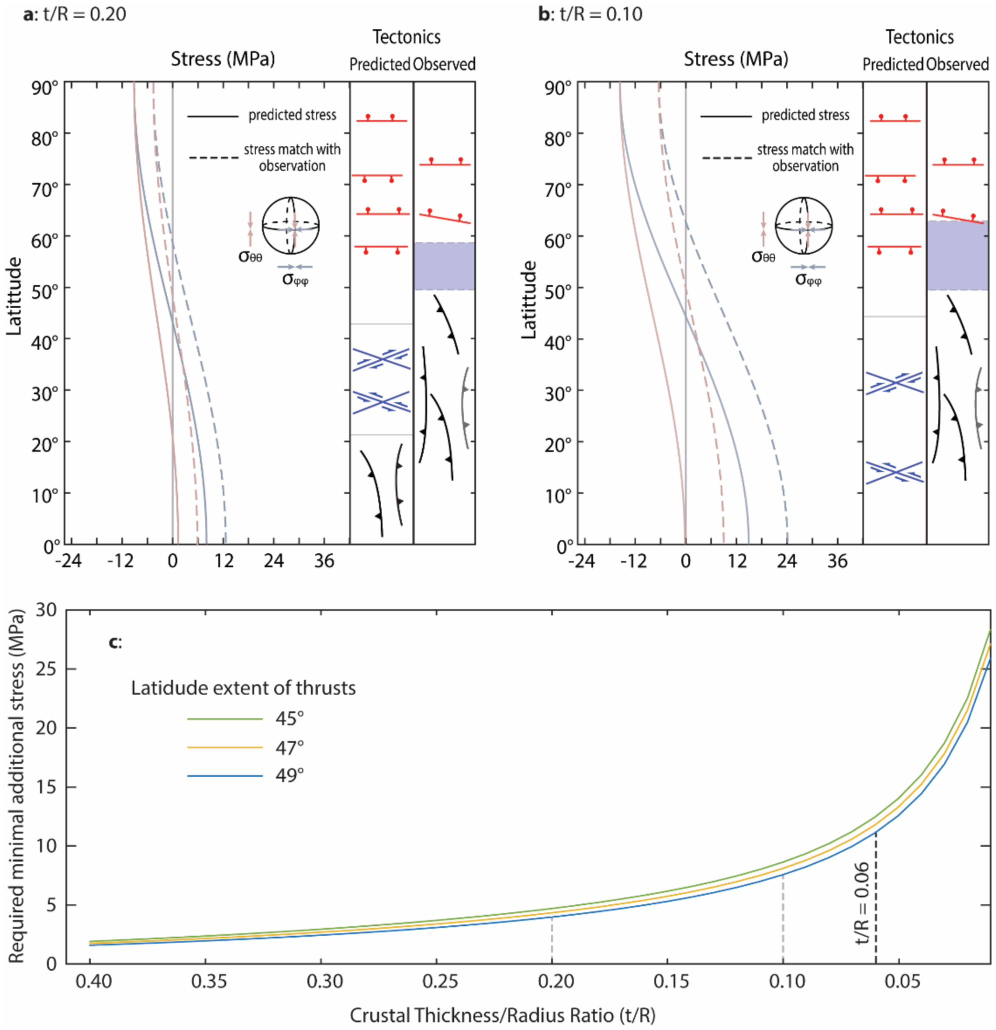

Fig. S3 Calculated stress field at different crustal thickness and corresponding additional stress to match with observation. Examples of crustal thickness at 120 km (a) and 60 km (b) present the variation in calculated stress (solid curves) and corresponding tectonic patterns. The stress field required to match with the observed extent of tectonic provinces (dashed lines). (c) plot of crustal thickness to radius ratio and required minimum additional stress at different scenarios of thrust distribution, with latitudinal extent to 45° (green), 47° (yellow), and 49° (blue). The crust to radius ratio of 0.20 (grey dash line), 0.10 (grey dash line), and 0.06 (black dash line) correspond to the three cases shown in panel a, b and in Fig. 5.

References:

1. Moore, J. M., McKinnon, W. B., Spencer, J. R., Howard, A. D., Schenk, P. M., Beyer, R. A., Nimmo, F., et al. (2016). The Geology of Pluto and Charon Through the Eyes of New Horizons. *Science, 351*(6279), 1284-1293. https://10.1126/science.aad7055
2. Schenk, P., Beyer, R. A., McKinnon, W. B., Moore, J. M., Spencer, J. R., White, O. L., New Horizons Geology and Geophysics Investigation Team, et al. (2018). Breaking Up is Hard to Do: Global Cartography and Topography of Charon from New Horizons. *Icarus, 315,* 124-145. https://doi.org/10.1016/j.icarus.2018.06.010
