# Supplementary material for: Early tidal despinning history recorded in the tectonics of Oz Terra, Charon
Source: Nat Commun. 2026 Jul 14;17:5978. doi: 10.1038/s41467-026-75069-7 (PMC13369154; doi:10.1038/s41467-026-75069-7)
Supplement: Supplementary file 2 — Transparent Peer Review file [file 41467_2026_75069_MOESM2_ESM.pdf]

nature portfolio

Peer Review File

Early Tidal Despinning History Recorded in the Tectonics of Oz Terra, Charon

Corresponding Author: Dr Hanzhang Chen

Parts of this Peer Review File have been redacted as indicated to remove third-party material.

This file contains all reviewer reports in order by version, followed by all author rebuttals in order by version.

Version 0:

Reviewer comments:

Reviewer #1

(Remarks to the Author)
This work is of extremely high quality and is strongly recommended for publication with minor revisions. The text is free from nearly all forms of minor faults and is proofed to a very high degree. The approach is novel and of unique scientific value. The methods are solid, well-presented, and make a convincing case from observations to conclusions. All figures are appropriate, accurate, and well-explained. The result is timely and important, as a possible first case of despinning evidence preserved on an ancient global terrain. A few relatively minor concerns are noted below, mainly with the selection and justification of inputs to the model, and not the model itself nor its final interpretation. The concerns noted are similar to issues encountered by many geology papers, and not unique to this work by any means. Therefore, it is expected to be resolved with ordinary adjustments to the wording.

The authors conclude with a signal (in shell thickness best-fit) that is sufficiently strong, that even if it were off by say a factor of 2-3, it would still represent a reasonable preference for the stated conclusions, in particular of a colder start than other reports have shown. This strong signal in the results helps to mitigate any worries for minor numerical assumptions.

Geologic interpretation on Charon is of course challenged by limited data, and the authors have done an excellent job of making the most out of the resolution, spatial coverage, and single-angle-of-illumination available.

Specific Notes:

Figure 1: The sentence noting the meaning of grey arrows is repeated in the caption. One instance can be removed.

A primary concern for this work regards the selection of features to be considered as candidate faults. Figure 1 is open to considerable differences in geologic interpretation, particularly as to what landform edges are included for study. It is naturally a longstanding and universal concern for geology papers, that human subjective interpretation of complex landforms has always been an issue. (Ideally someday in the future, machine-learning based edge-detection algorithms might be standardized enough to allow less subjective determination of what is declared an arcuate/lobate feature and what is not. However, this manuscript must not be held to any higher standard of proof than all previous literature, and thus human subjectivity remains the widespread baseline for such works.)

In effect this is asking the authors to not only justify the positive selection of candidate features declared to be arcuate scarps of any confidence level, but also to justify the negative side as well. This means, briefly explain why visibly-apparent semi-arcuate or semi-linear features visible in Fig. 1 that are not labeled with grey or white arrows, are deemed by the authors as not being candidates. Many possible reasons come to mind (e.g. features already studied in other literature as having a distinctly different origin, which is perhaps not in conflict with the study here). But it is best for the authors to state their own case-by-case logic.

In particular, the following un-labeled features should be briefly explained for their non-selection, because they appear to go against the hypothesis of north-south orientation predominance:

- The lower righthand quadrant of Fig. 1 is filled with multiple parallel east-west linear elevation change features.

- Starting from the topographic local minimum of the deep blue circle in the upper left, trending diagonally to the upper right corner of Fig. 1, can be interpreted as a lengthy linear feature of mostly east-west orientation.

- Likewise, an east-west feature (almost exactly parallel to that noted just above) spanning all of Fig. 1 appears to start from the lower lefthand corner, traversing to the midpoint of the righthand edge (with some interruption). Again, please note in the text why this feature, or segments of it, are not included for study here.

- Fig. 1 and Oz Terra in general could be interpreted as being broken up into several large massifs or elevated landform blocks, about 6-10 in total, each roughly rectangular. But by such interpretation, edges visibly seem to exist on all sides, north, south, east and west combined.

-The northernmost 'hook' of the scarp containing profile B-B', seems to have about equal visible confidence as an alternate path trending to the west from the point where B-B' crosses. The alternate path would appear less like a north-south overall pattern, and more like a perimeter to a regional upland massif block, (as the scarp containing A-A' already appears). Please justify this and similar choices in the text.

- At about 50 deg north and between 15 deg W and 0 deg, there exists a linear feature which likewise appears to be lobate in form, yet is mainly east-west in orientation. This feature is clearly not a remnant crater rim. Again, it appears as the lower perimeter of an upland massif. Please note in text why this feature is decided not a candidate for study here.

The authors are encouraged to label the noted scarps, so that they can easily be referred to individually by readers.

The authors are urged to change language regarding the use of an elastic model in this work. The manuscript first states that craters a and b appear to have 'negligible' relaxation. However, again, this term is open to considerable subjective interpretation, and both of these craters could just as well be described has having a degree of relaxation about halfway from appearing fresh, to the opposite extreme of becoming largely erased by relaxation alone (or thus appearing 'halfway relaxed' or 'at a medium degree or relaxation' or 'at a degree of relaxation that is somewhere between end-member extremes'. This is partly a problem of having limited immediate comparisons to work from. The authors are again commended for doing the best one can with limited image data.) This wording would be of little concern, except that it is used later to justify the elastic model choice. I would urge the authors to change how this is achieved:
- A primary reason for using an elastic model, is simply that such studies must begin somewhere, and an elastic model is almost always the appropriate first choice, especially in the outer solar system. In fact, there is high value for studies to begin by publishing elastic results, to serve as an invaluable baseline against which any non-elastic future investigations must be compared.
- If the relaxation of the total field of Oz Terra were felt by any reader to be 'moderate' instead of 'negligible', the elastic choice and cascading results would thus seem weakened to that reader.
- There are many reasons why a non-elastic model should be tested by future work (after this work is published). Among them are the point that the basal temperature of the ice layer is substantially higher than the surface temperature, with brittle elastic behavior often apparent at the surface, including the ability to support sharp topography long-term, yet the majority of the ice thickness can still best be considered ductile. (Certainly this work does not need to include such an upgrade, yet the authors are of course encouraged to consider this for their own future research directions.)
- As the viscous regime is also a function of stress magnitude, it thus can vary with time, depth and laterally within a single fault system. Ideally, the authors are urged to include in the text, the anticipated creep regime based on both thermal state and deviatoric stress, particularly for conditions expected around ~4.0 Ga on Charon. This is expected to help justify the elastic assumption used.
- Therefore in total, wording is recommend akin to "an elastic model is assumed for investigation based on surface relaxation that is not severe, as well as the literature standard of utilizing an elastic baseline in the absence of pronounced or obvious viscous requirements. Future work will no doubt test this assumption using increasingly detailed viscoelastic models including temporal-spatial variation of controlling parameters."

Akin to the concern for subjective case selection for scarps overall, is the concern for profile selection of A-A' and B-B'. Please briefly note why these two profiles are chosen for reporting, above all other possible slices. (E.g. it seems A-A' was chosen because it contains a topographic highpoint, for maybe better signal to noise?) B-B' seems moderately surprising, because it is not drawn exactly perpendicular to the local scarp proposed orientation and crosses a possible secondary ridge tail in the background terrain. This however may be a purposeful demonstration that the model can match even off-perpendicular and off-ideal slices, and if this was done for this reason, briefly state so.

Ideally, if the authors have tested further fits beyond A-A' and B-B', this may be included in the manuscript without compromising total length, simply by noting, if true, something like "Numerous additional profiles were tested for fit and found qualitatively indistinguishable to those highlighted in Fig. 2."

Fig. 3 labeling and caption: It seems notable that what is referred to as 'knob-bearing terrain' is also 'groove-bearing terrain', as none of the labeled and visible grooves appear in the trough-bearing terrain. The authors may find it useful to revise to reflect that grooves, maybe more than knobs, set this area apart.

Regarding craters a and b, the authors do an excellent job of considering the implications of observations, as well as

including detailed comparisons to other worlds, exactly as hoped for in such circumstances. The alternate hypothesis of mantling infill is appropriately considered and noted. A few adjustments in wording however would perhaps strengthen the authors' case. Crater a's missing half actually does have a faint visible remnant, suggesting it was not totally 'obliterated', as described in text. A further alternate hypothesis is that crater a formed on top of a pre-existing division of landforms, and the degraded half retains far less profile because either it was a severe lowland to begin with and never built any sharp eastern wall, or that it was a warmer, more damaged, or more compositionally distinct terrain type that degraded faster.

Just southeast of crater a on the same candidate scarp, and just outside the southeast edge of the boundary of Fig 3, is a third crater that would seem to also have the same cross-cutting as crater b, with the scarp grazing the edge. Please note if this was considered, and if this cater was omitted from study for simple reasons such as lacking sufficient image resolution.

The overall case for the relative timing for craters a and b and tectonic activity is well-justified in the text.

The sentences highlighting that features are unlikely to be the result of any single seismic event, and are almost certainly the result of many events, could be removed to accommodate some length for other clarifications. The possibility of such features from one seismic event ~4.0 Ga is so unlikely that it could safely go without saying.

Section 6:
The authors note that the fit of features indicates limited true polar wander. However, this seems to invert the dependency. It would seem instead that achieving a fit is instead predicated upon a starting assumption that true polar wander is limited.

For the clause, 'resulted from recession', please clarify, 'orbital recession'?

If results are a full order of magnitude greater than inferred from flexure, please briefly note in text, such as with one sentence, why the flexure results might be so far off?

It seems incongruous that for the nomenclature in the Charon literature, 'cold start' is connected to the giant impact hypothesis. For other worlds, 'cold start' is reserved for situations with very slow accretion, heat escape prior to burial, and a lack of any massive thermal event later such as a giant impact. How is a giant impact not a 'warm start', or 'warm reset', relative to even colder/more-quiescent alternates? (e.g., is the 'cold start' of a Charon giant impact actually referring to the 'start' being long before the giant impact occurred? And if so, wouldn't its traces, at least upon shell depth, not be reset by the impact?) While the cited literature likely explains this at length not available here, it would be best to help readers understand this manuscript's final and strongest conclusion, by incorporating just one brief sentence explaining this seeming incongruity in the nomenclature.

Modeling and equations:
The model is well-presented in fully appropriate detail. The model parameters are well presented in table format and the numerical values for those items subject to author choice are well-chosen. Software and data sources are well-described. No concerns with the numerical modeling are noted. Figures 4, 5 and 6 are robust and clear. The method used is novel, by using the terrain positive deformation fit as a fully reasonable approach to constraining subsurface geometry. There is some concern that the fit might be influenced as much by other parameters as the two chosen for primary study, but this is a minor worry, and such multidimensional equation systems must limit fitting somehow, and the relative parameter sensitivities are well enough addressed to make the needed case here.

Overall, the possibility that Charon is indeed a case of expression of the Voyager-era despinning surface predictions, is a highly valuable study and a possible landmark determination. The authors are commended for a well-presented and logical chain of inferences from limited surface imagery, using a robust model approach, to new constraints on formation scenarios. Again, the only strong concern is just with improving justification of what linear features are chosen or set aside, based on their apparent topography. I look forward to seeing this work with such minor adjustments and then soon seeing its eventual publication.

Reviewer #2

(Remarks to the Author)
This manuscript argues that arcuate positive relief ranges in Charon's Oz Terra are compressional structures formed by thrusting and that their distribution, combined with other tectonic trends, records an early tidal despinning signal. The authors fit two topographic swath profiles with an elastic dislocation thrust model to infer fault geometry and a minimum elastic shell thickness of roughly 30 to 36 km, then convert inferred shortening into about 1 percent equatorial compression and an implied early rotation period near 14 hours. They compare predicted despinning stress provinces to mapped tectonics and invoke an added global contraction component to better match the observed latitude extent of compression. I recommend publication with revisions.

The logic mostly progresses in a clear sequence: mapping and interpretation of the arcuate ranges, mechanical inversion for fault geometry, conversion of shortening to regional strain, conversion of strain to flattening and initial spin, then comparison of predicted stress provinces to observations. The main intellectual jumps occur where local measurements are scaled to global quantities and where additional stress terms are introduced to reconcile mismatches.

Major comments:

The manuscript makes a plausible thrust interpretation, but it moves quickly from “arcuate positive relief” to “compressional thrusting” without a fully explicit elimination of other mechanisms. The authors do acknowledge normal faulting as less likely in one key place, which helps, but the review would be stronger with a more structured argument for what observations are uniquely diagnostic of thrusting and what observations would be expected under competing hypotheses.

Non uniqueness of the inversion and the mapping from fault root depth to elastic thickness
Okada style dislocation fits can be non-unique, with trade offs among dip, depth, slip magnitude, and slip distribution. The manuscript reports best fits and uses inferred root depth as a lower bound on elastic thickness, but the text does not always adequately discuss the extent of any non-uniqueness in the solution. In addition, treating the fault root depth as the detachment depth for balanced cross section shortening is a substantive assumption. Mechanical stratification could allow detachments above or below the effective elastic thickness. This does not invalidate the approach, but it should be framed as an estimate with wider uncertainty and supported a discussion of sensitivities and uncertainties in the solution.

To match the observed latitude extent of thrust tectonics, the authors add an additional compressive stress of about 20 MPa attributed to global contraction. This is plausible, but it is introduced primarily to reconcile a mismatch between prediction and observation. The argument would be more convincing if the contraction magnitude were constrained independently, for example via thermal evolution or porosity closure estimates, or if the paper showed that a reasonable range of contraction magnitudes that still yield the correct tectonic province pattern.

Stress province comparison would benefit from more explicit mapping uncertainty
The discussion notes discrepancies such as the absence of predicted mid latitude strike slip systems. This is a useful observation. It would be strengthened by clarifying whether strike slip absence is a data coverage limitation, a mapping completeness issue, or a true geologic absence that challenges the model assumptions.

Version 1:

Reviewer comments:

Reviewer #2

(Remarks to the Author)
I have no further comments. The authors addressed all points raised.

Open Access This Peer Review File is licensed under a Creative Commons Attribution 4.0 International License, which permits use, sharing, adaptation, distribution and reproduction in any medium or format, as long as you give appropriate credit to the original author(s) and the source, provide a link to the Creative Commons license, and indicate if changes were made.
In cases where reviewers are anonymous, credit should be given to 'Anonymous Referee' and the source.
The images or other third party material in this Peer Review File are included in the article’s Creative Commons license, unless indicated otherwise in a credit line to the material. If material is not included in the article’s Creative Commons license and your intended use is not permitted by statutory regulation or exceeds the permitted use, you will need to obtain permission directly from the copyright holder.

To view a copy of this license, visit https://creativecommons.org/licenses/by/4.0/

Response to Reviewer comments for manuscript NCOMMS-25-88493-T

Please see the response to the review comments accompanying the revised manuscript titled '*Early Tidal Despinning History Recorded in the Tectonics of Oz Terra, Charon*' submitted for publication as a research article in *Nature Communications*.

We would like to thank the two reviewers for their insightful and constructive comments, which significantly improved the clarity and presentation of the manuscript. Reading through the reviewer's comments, we find common concerns and suggestions: (1) the need for a clearer reasoning on the thrust interpretations for arcuate ranges with comparison to the extensional features and (2) the need to strengthen the modeling part by providing justification of the model assumptions and incorporating discussions on both the uncertainties in model inputs and outputs.

We carefully address the reviewers' comments. We list the major changes below in response to the shared concerns and indicate the relevant revised figures and sections. We also reply point by point to the detailed comments in blue (referred to by the reviewer number and comment number, i.e., R1C1).

Our major changes fall into two categories: i) justification on the selection of arcuate range and its interpretation as thrust fault interpretation by specifying and documenting the criteria for separating compressional features from extensional features, ii) improve the modeling description and discussion by providing the justification of basic assumptions, adding discussions on the uncertainties caused by the model parameterization and their corresponding influence on the expected tectonic features and require stress estimation. We revised the main text and figures as necessary (e.g., Fig. 1 and Fig. 3) and added sections to methods to address the concerns, including 2 new figures (Fig. S1, S3) and one table (Table S1).

We believe that these revisions substantially strengthen the manuscript from the prior submission, clarifying the robustness and limitations of our approaches. Some of the figures and discussion are only included in this response but not in the revised manuscript due to space limitations and readability. Please see below a detailed summary list of what we have specifically addressed:

i) Added descriptions of large-scale geomorphic features and a summary of selected arcuate ranges ([R1C2] and [R2C1])

a) We generated a slope map to objectively identify linear topographic features (Fig. S1),
b) We specified the selection criteria for arcuate ranges with a table comparing the topographic characteristics of compressional features (arcuate ranges) and extensional features (Table S1).
c) We labeled the candidate arcuate ranges (*I-V*) and other groups of linear features (*a-d*) for easier reference and explained, case by case, for the non-selected groups.
d) These changes are provided in the newly added method section titled Topographic Analysis and Identification of Candidate Arcuate Ranges.

ii) Expanded discussion on the uncertainties in the modeling results and tectonic implications in the Discussion ([R1C3], [R1C13], [R2C2], and [R2C3])

e) We explored the influence of changing elastic thickness on the predicted stress fields and presented two examples (Fig. S3 a and b),
f) We calculated the required minimal additional stress to reconcile model prediction and observed tectonic pattern for the ranges of elastic thickness and included the uncertainty of the interpreted latitudinal extent of tectonic features (Fig. S3 c).
g) We discussed the uncertainty caused by trade-offs between model input parameters of the Okada model and presented the associated uncertainties in elastic thickness and stress estimates (method section: Uncertainties in elastic modeling and inferred stress estimates).

Please see the detailed, point-by-point responses to each reviewer below.

REVIEWER COMMENTS

Reviewer #1 (Remarks to the Author):

This work is of extremely high quality and is strongly recommended for publication with minor revisions. The text is free from nearly all forms of minor faults and is proofed to a very high degree. The approach is novel and of unique scientific value. The methods are solid, well-presented, and make a convincing case from observations to conclusions. All figures are appropriate, accurate, and well-explained. The result is timely and important, as a possible first case of despinning evidence preserved on an ancient global terrain. A few relatively minor concerns are noted below, mainly with the selection and justification of inputs to the model, and not the model itself nor its final interpretation. The concerns noted are similar to issues encountered by many geology papers, and not unique to this work by any means. Therefore, it is expected to be resolved with ordinary adjustments to the wording.

The authors conclude with a signal (in shell thickness best-fit) that is sufficiently strong, that even if it were off by say a factor of 2-3, it would still represent a reasonable preference for the stated conclusions, in particular of a colder start than other reports have shown. This strong signal in the results helps to mitigate any worries for minor numerical assumptions.

Geologic interpretation on Charon is of course challenged by limited data, and the authors have done an excellent job of making the most out of the resolution, spatial coverage, and single-angle-of-illumination available.

We thank the reviewer for the positive and constructive feedback and for acknowledging the limited remote-sensing data and geologic interpretation in planetary geology. We have carefully revised the manuscript in response to your thoughtful suggestions to further enhance the clarity and rigor of the presentation. Please see below the response to each of the comments.

Specific Notes:

[R1C1] Figure 1: The sentence noting the meaning of grey arrows is repeated in the caption. One instance can be removed.

Thank you for the careful proofreading. We have removed the repeated parts in the figure caption of Figure 1.

[R1C2] A primary concern for this work regards the selection of features to be considered as candidate faults. Figure 1 is open to considerable differences in geologic interpretation, particularly as to what landform edges are included for study. It is naturally a longstanding and universal concern for geology papers, that human subjective interpretation of complex landforms has always been an issue. (Ideally someday in the future, machine-learning based edge-detection algorithms might be standardized enough to allow less subjective determination of what is declared an arcuate/lobate feature and what is not. However, this manuscript must not be held to any higher standard of proof than all previous literature, and thus human subjectivity remains the widespread baseline for such works.)

In effect this is asking the authors to not only justify the positive selection of candidate features declared to be arcuate scarps of any confidence level, but also to justify the negative side as well. This means, briefly explain why visibly-apparent semi-arcuate or semi-linear features visible in Fig. 1 that are not labeled with grey or white arrows, are deemed by the authors as not being candidates. Many possible reasons come to mind (e.g. features already studied in other literature as having a distinctly different origin, which is perhaps not in conflict with the study here). But it is best for the authors to state their own case-by-case logic.

In particular, the following un-labeled features should be briefly explained for their non-selection, because they appear to go against the hypothesis of north-south orientation predominance:

We thank the reviewer for raising this concern. We acknowledge that subjective interpretation is inevitable in the process of geologic mapping. We agree that the limited data in planetary geology made it even harder to interpret certain structures. We acknowledge that we need to 1) improve the description of our selection of faults and 2) explain the other features that were mapped and interpreted in previous literature. To address this, we now include the description of existing features in Fig. S1 along with Table S1. We generated a slope map (Fig. S1) of the research area. Taking both slope and morphologic characteristics into consideration, we presented a more objective and specific criteria to identify potential arcuate ranges. We now divide linear features into two types, scarps and arcuate ranges. Table S1 summarizes the geomorphic characteristics of these landforms of arcuate ranges and scarps. See added description in the Method section (Topographic Analysis and Identification of Candidate Arcuate Ranges) and lines 82 - 86 in the main text: *"We performed systematic analysis of landforms in the low to mid-latitudes of Oz Terra, Charon's northern highland, and identified large-scale (> 10s km) topographic features that can be divided into two groups (see Methods). The arcuate ranges on Charon appear as positive relief landforms with characteristic morphology, which are different from other large-scale landforms (arcuate range I-V in Fig. 1, Methods)."*

Added "Section 8.2 Topographic Analysis and Identification of Candidate Arcuate Ranges"

We examined the topography of the entire Oz Terra region and comprehensively identified large-scale linear features by overlaying the digital elevation model (DEM) on a DEM-derived slope map (Fig. S1).

We consider linear features with length greater than 50 km, where elevation change matches with >15° slopes. These features can be generally divided into two types: (type 1) steep slopes (>35°), large elevation change (>3 km), and short-length segments (~ 50 km); (type 2) moderate slopes (<35°), small elevation change (<3 km), and long lengths (>200 km). Previous mapping works [13,42] captures all the type 1 features (e.g., feature a-d in Fig. S1), but missing several of type 2 features (e.g., feature I, IV, V in Fig. S1).

Based on the morphology of sharp crest lines adjacent to steep planer slopes, type 1 features were interpreted as normal fault scarps [10,13,42], which is further supported by the observation of extensive graben-like troughs. The moderate slopes and missing sharp crest lines of type 2 features seems to contradict with an interpretation of normal fault scarps. Instead, their correlation with ranges and arcuate traces resembles topography of thrust-origin landforms. The contrasting morphologic criteria used to distinguish these two landform groups are summarized in Table S1.

Examples of Type 1 include linear or lobate scarp features that can be readily identified in the slope map (a – d in Fig. S1). Group a is a series of tectonic features trending northeast, often referred to as the tectonic belt [10]. These features are characterized by steep scarps (>35°) and often host sharp crests (Fig. 1), which are typical of normal-fault-related landforms. Their planar slopes and concave-toward-lowland morphology further support this interpretation. Group b consists of a series of northeast-trending broad troughs in the mid-latitude region. The traces marking the elevation change are relatively straight rather than arcuate in shape and often occur in pairs bounding the trough. Although neither distinct planar scarps nor sharp crest ridge lines are developed, an arcuate range interpretation is less likely because thrusts rarely develop in paired configuration opposing one another. Group c comprises a set of sub-parallel northwest-trending scarps adjacent to the tectonic belt. These features show linear or southward-concave sharp crest lines consistent with a normal-fault origin. Feature d appears as an east-trending arcuate front with a rounded range top. This feature however is connected to a concave, east-trending planar scarp, with a steeper slope that bounds part of Group b. Thus, feature d is considered a less likely candidate for an arcuate range.

We identified Type 2 features based on their morphologic characteristics (Table S1) and selected them as potential thrust-induced arcuate ranges. Arcuate ranges I and II exhibit gentle frontal slopes that are convex toward the adjacent lowlands and have rounded range tops. Arcuate range III shows similar characteristics but has steeper frontal slopes. Arcuate range IV and V also appear as convex features, marking the abrupt termination of other landform features. However, the morphologic patterns along these two traces are less pronounced than along the others. Arcuate range I and II are selected for topographic analysis, and we extracted two topographic profiles (A-A' and B-B' in Fig. 1). We selected range I and II because they are located within relatively higher resolution DEM regions (e.g., ~ 400 m) [24]. They have pristine, characteristic asymmetric topographic profiles that are not affected by post cratering, and the similar reference elevation in the foreland and hinterland. We did not include arcuate range III due to a low reference elevation in its foreland, potentially due to the juxtaposition with extensional features of group b. The location of arcuate range I, II, and IV are considered to estimate the spacing between arcuate ranges for shortening calculation (see details in Methods: Despinning-induced Stress Field Calculation).

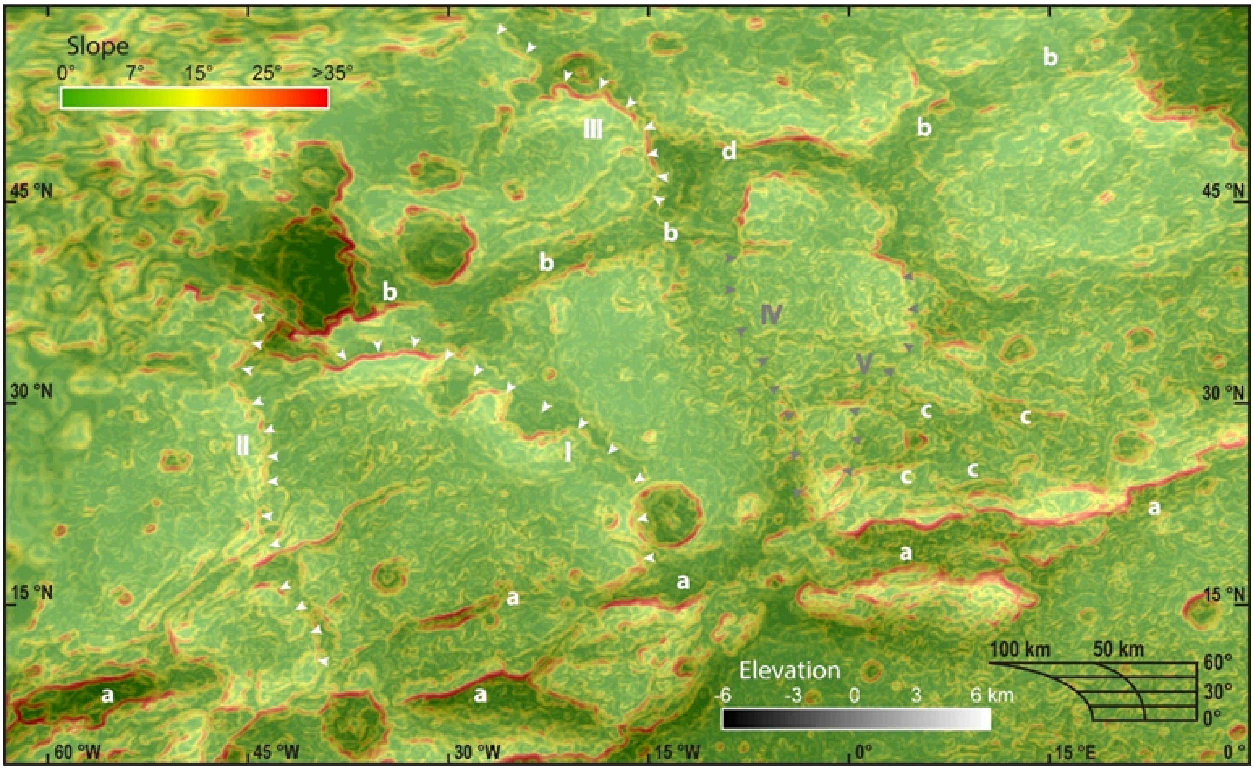

Fig. S1 Slope map of the study area. The map shows the color-coded slope value overlying the digital elevation model of the study area. *Type 1* features are labeled in (*a*~*d*), which are more likely extension-related features. The white and grey arrows trace out *Type 2* features (*I*~*V*), same as the potential arcuate range features presented in Fig. 1.

**Landforms**	***Type 1*** **Scarps**	***Type 2*** **Arcuate ranges**
**Topographic lengths**		
- **Along-strike**	- ~50 km	- >200 km
- **Cross -strike**	- ~10 km	- ~30 – 50 km
**Map view traces**	straighter and in segments	acuate
**Topographic relief**	>3 km	<3 km
**Range front slopes**	>35°	<35°

**Slope morphology**	concave upward<br>toward lower elevations	convex upward<br>toward lower elevations
**Ridge crest morphology**	sharp ridge crest lines	broad, rounded ridge tops
**Interpreted fault types**	normal faults	thrust faults

- The lower righthand quadrant of Fig. 1 is filled with multiple parallel east-west linear elevation change features.

We now labeled these features as Type 1, group *c* in Figure S1, and included in the geomorphic feature descriptions. They commonly exhibit sharp ridge crests and planar slopes, which were interpreted as normal fault scarps in previous papers (e.g., Beyer et al., 2017). They are mostly concave towards the lower elevation within each segment. To clarify, we now add text from lines 388 - 390: "*Group c comprises a set of sub-parallel northwest-trending scarps adjacent to the tectonic belt. These features show linear or southward-concave sharp crest lines consistent with a normal-fault origin.*"

- Starting from the topographic local minimum of the deep blue circle in the upper left, trending diagonally to the upper right corner of Fig. 1, can be interpreted as a lengthy linear feature of mostly east-west orientation.

We now labeled these features as Type 1, group b in Figure S1. These features are broad grabens without sharp bounding scarps. We ruled out these features mainly because thrust faults are unlikely to develop in pairs against each other to form the observed symmetric troughs. To clarify, we added text from lines 383 - 388: "*Group b consists of a series of northeast-trending broad troughs in the mid-latitude region. The traces marking the elevation change are relatively straight rather than arcuate in shape and often occur in pairs bounding the trough. Although neither distinct planar scarps nor sharp crest ridge lines are developed, an arcuate range interpretation is less likely because thrusts rarely develop in paired configuration opposing one another.* "

- Likewise, an east-west feature (almost exactly parallel to that noted just above) spanning all of Fig. 1 appears to start from the lower lefthand corner, traversing to the midpoint of the righthand edge (with some interruption). Again, please note in the text why this feature, or segments of it, are not included for study here.

We labeled these features as Type 1, group a in Figure S1. These features exhibit sharp ridge crests, planar slopes, and are sometimes distributed in pairs, bounding trough morphology. This belt of features was referred to as the tectonic belt in previous Charon geology papers (e.g., Moore et al. (2015) [10] and Beyer et al. (2017) [13] in the reference list). To clarify, we added text from lines 379 - 383: "*Group a is a series of tectonic features trending northeast, often referred to as the tectonic belt [10]. These features are characterized by steep scarps (>35°) and often host sharp crests (Fig. 1), which are typical of normal-fault-related landforms. Their planar slopes and concave-toward-lowland morphology further support this interpretation.* ".

- Fig. 1 and Oz Terra in general could be interpreted as being broken up into several large massifs or elevated landform blocks, about 6-10 in total, each roughly rectangular. But by such interpretation, edges visibly seem to exist on all sides, north, south, east and west combined.

We acknowledge that some of the landforms are interpreted as broken landform blocks by the Beyer et al. (2017). However, we found that the surface morphology differs in adjacent blocks and the morphological characteristics of 'edges' vary (e.g., slope, crest line, map view traces). In the slope map of Fig. S1, the slope value varies drastically from >35° to <15° along those proposed boundaries. The arcuate range landforms that we interpret show different morphologies of normal fault scarps bounding the proposed intact crustal blocks. To address this concern, we now add text in line 123-127 to clarify this interpretation: "*Though east-dipping normal faulting is capable of generating the observed vertical offset, it is a less likely mechanism, as the anticipated normal fault scarps are not observed, while the range front is arcuate instead of the anticipated polygonal geometry in the proposed intact primordial crustal blocks model [10].*"

-The northernmost 'hook' of the scarp containing profile B-B', seems to have about equal visible confidence as an alternate path trending to the west from the point where B-B' crosses. The alternate path would appear less like a north-south overall pattern, and more like a perimeter to a regional upland massif block, (as the scarp containing A-A' already appears). Please justify this and similar choices in the text.

In optical images (i.e., Fig. R1 presented below), the trace of *arcuate range II* appears to extend towards northeast rather than going northwest. Besides, we can see another set of linear features trending NW that may have caused the elevation change that the reviewer pointed out. However, these features are beyond the coverage of higher-quality parts of the digital elevation model (see Fig. R2).

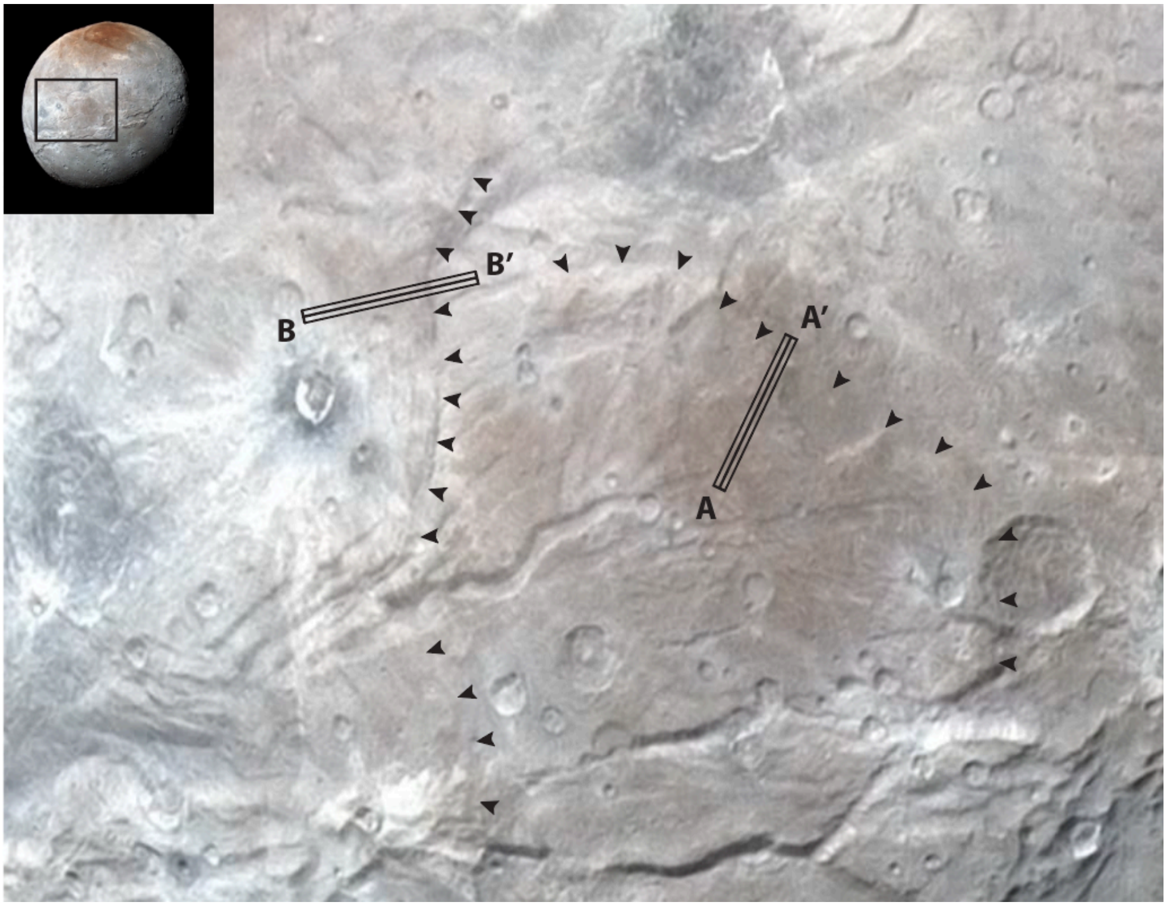

Figure R1. Optical image of the area around arcuate range II. Arcuate ranges *I* and *II* are marked by arrows. Locations of topographic swath profiles A-A' and B-B' are given for reference. The image is cropped from the enhanced color image of Charon (Photo ID: PIA19968).

- At about 50 deg north and between 15 deg W and 0 deg, there exists a linear feature which likewise appears to be lobate in form, yet is mainly east-west in orientation. This feature is clearly not a remnant crater rim. Again, it appears as the lower perimeter of an upland massif. Please note in text why this feature is decided not a candidate for study here.

We labeled this feature as Type 1, feature *d* in Fig. S1. This lobate feature appears to be a northern bounding scarp of a broad east-west trending trough. Or, in Beyer et al., (2017), a southern bounding scarp of a 'crustal block'. Thus, less likely to be an arcuate range. To clarify, we added text from line 366 - 369.

The authors are encouraged to label the noted scarps, so that they can easily be referred to individually by readers.

We thank the reviewer for the suggestion. We updated Fig. 1, including labels *I-IV* for the arcuate ranges and added Fig. S1 to also show the labels for the non-selected feature groups *a-d*.

[R1C3] The authors are urged to change language regarding the use of an elastic model in this work. The manuscript first states that craters a and b appear to have 'negligible' relaxation. However, again, this term is open to considerable subjective interpretation, and both of these craters could just as well be described has having a degree of relaxation about halfway from appearing fresh, to the opposite extreme of becoming largely erased by relaxation alone (or thus appearing 'halfway relaxed' or 'at a medium degree or relaxation' or 'at a degree of relaxation that is somewhere between end-member extremes'. This is partly a problem of having limited immediate comparisons to work from. The authors are again commended for doing the best one can with limited image data.) This wording would be of little concern, except that it is used later to justify the elastic model choice. I would urge the authors to change how this is achieved:

We thank the reviewer for this comment on the language used to justify applying an elastic model to landforms on Charon. We changed the wording of 'negligible' to 'limited' in the text for a broader interpretation of the relaxation state of the craters, following your suggestions in the next comments.

- A primary reason for using an elastic model, is simply that such studies must begin somewhere, and an elastic model is almost always the appropriate first choice, especially in the outer solar system. In fact, there is high value for studies to begin by publishing elastic results, to serve as an invaluable baseline against which any non-elastic future investigations must be compared.
- If the relaxation of the total field of Oz Terra were felt by any reader to be 'moderate' instead of 'negligible', the elastic choice and cascading results would thus seem weakened to that reader.
- There are many reasons why a non-elastic model should be tested by future work (after this work is published). Among them are the point that the basal temperature of the ice layer is substantially higher than the surface temperature, with brittle elastic behavior often apparent at the surface, including the ability to support sharp topography long-term, yet the majority of the ice thickness can still best be considered ductile. (Certainly this work does not need to include such an upgrade, yet the authors are of course encouraged to consider this for their own future research directions.)
- As the viscous regime is also a function of stress magnitude, it thus can vary with time, depth and laterally within a single fault system. Ideally, the authors are urged to include in the text, the anticipated creep regime based on both thermal state and deviatoric stress, particularly for conditions expected around ~4.0 Ga on Charon. This is expected to help justify the elastic assumption used.
- Therefore in total, wording is recommend akin to "an elastic model is assumed for investigation based on surface relaxation that is not severe, as well as the literature standard of utilizing an elastic baseline in the absence of pronounced or obvious viscous requirements. Future work will no doubt test this assumption using increasingly detailed viscoelastic models including temporal-spatial variation of controlling parameters."

We thank the reviewer for the suggestion to change the justification for applying an elastic model here to a starting point for more sophisticated modeling work. We agree that an elastic model is a good starting point, but further modeling is needed for full consideration of various aspects (e.g., viscous relaxation, plastic deformation, etc.). We have now adopted the reviewers' suggested language and made some revisions. Please see revised text in lines 143 - 145: "*An elastic model is assumed for investigation based on*

surface relaxation that is not severe, as well as the literature standard of utilizing an elastic baseline in the absence of pronounced or obvious viscous requirements."

[R1C4] Akin to the concern for subjective case selection for scarps overall, is the concern for profile selection of A-A' and B-B'. Please briefly note why these two profiles are chosen for reporting, above all other possible slices. (E.g. it seems A-A' was chosen because it contains a topographic highpoint, for maybe better signal to noise?) B-B' seems moderately surprising, because it is not drawn exactly perpendicular to the local scarp proposed orientation and crosses a possible secondary ridge tail in the background terrain. This however may be a purposeful demonstration that the model can match even off-perpendicular and off-ideal slices, and if this was done for this reason, briefly state so.

We thank the reviewer for raising the problem of selection, which will improve this manuscript. We select these two profiles mainly because of better mosaic resolution and DEM quality at these two locations (Fig. R2). The profile A-A' partially falls into the highest-resolution and best-phase angle strip of images taken for Charon. The profile B-B' falls into the area with decent image resolution and good phase angle.

The intended location of profile B-B' is to avoid the crater in the hinterland and east-trending troughs in the foreland (Fig. 1). The thrust belt where the profile B-B' crossed appears in segments, as you can see the positive-topography belt consists of 2-3 segments of eastward-convex arcs and overprinted by the troughs in its southern section (Fig. 1 and Fig. R1). In this case, the orientation of 'exact' perpendicular is hard to assess.

[REDACTED]

Figure R2. Modified from Schenk et al., (2018). The study area presented in Figure 1 is overlapped onto the global image resolution map (upper) and image phase angle map (lower) of Charon.

Ideally, if the authors have tested further fits beyond A-A' and B-B', this may be included in the manuscript without compromising total length, simply by noting, if true, something like "Numerous additional profiles were tested for fit and found qualitatively indistinguishable to those highlighted in Fig. 2."

We thank the reviewer for the suggestion. We did attempt to model profiles from other arcuate ranges. However, the two profiles that were presented are the most characteristic range profiles. Others may have been modified by other processes (e.g., cratering) or not in identical asymmetric ridge shape.

We have now added the following sentences clarifying the use of various profiles from line 403 to line 410: "*We selected range I and II because they are located within relatively higher resolution DEM regions (e.g., ~ 400 m) [24]. They have pristine, characteristic asymmetric topographic profiles that are*

not affected by post cratering, and the similar reference elevation in the foreland and hinterland. We did not include arcuate range III due to a low reference elevation in its foreland, potentially due to the juxtaposition with extensional features of group b. The location of arcuate range I, II, and IV are considered to estimate the spacing between arcuate ranges for shortening calculation (see details in Methods: Despinning-induced Stress Field Calculation)."

[R1C5] Fig. 3 labeling and caption: It seems notable that what is referred to as 'knob-bearing terrain' is also 'groove-bearing terrain', as none of the labeled and visible grooves appear in the trough-bearing terrain. The authors may find it useful to revise to reflect that grooves, maybe more than knobs, set this area apart.

We thank the reviewer for this comment. We updated the legend to include groove-bearing for the specific landform unit. Please see the updated Figure 3.

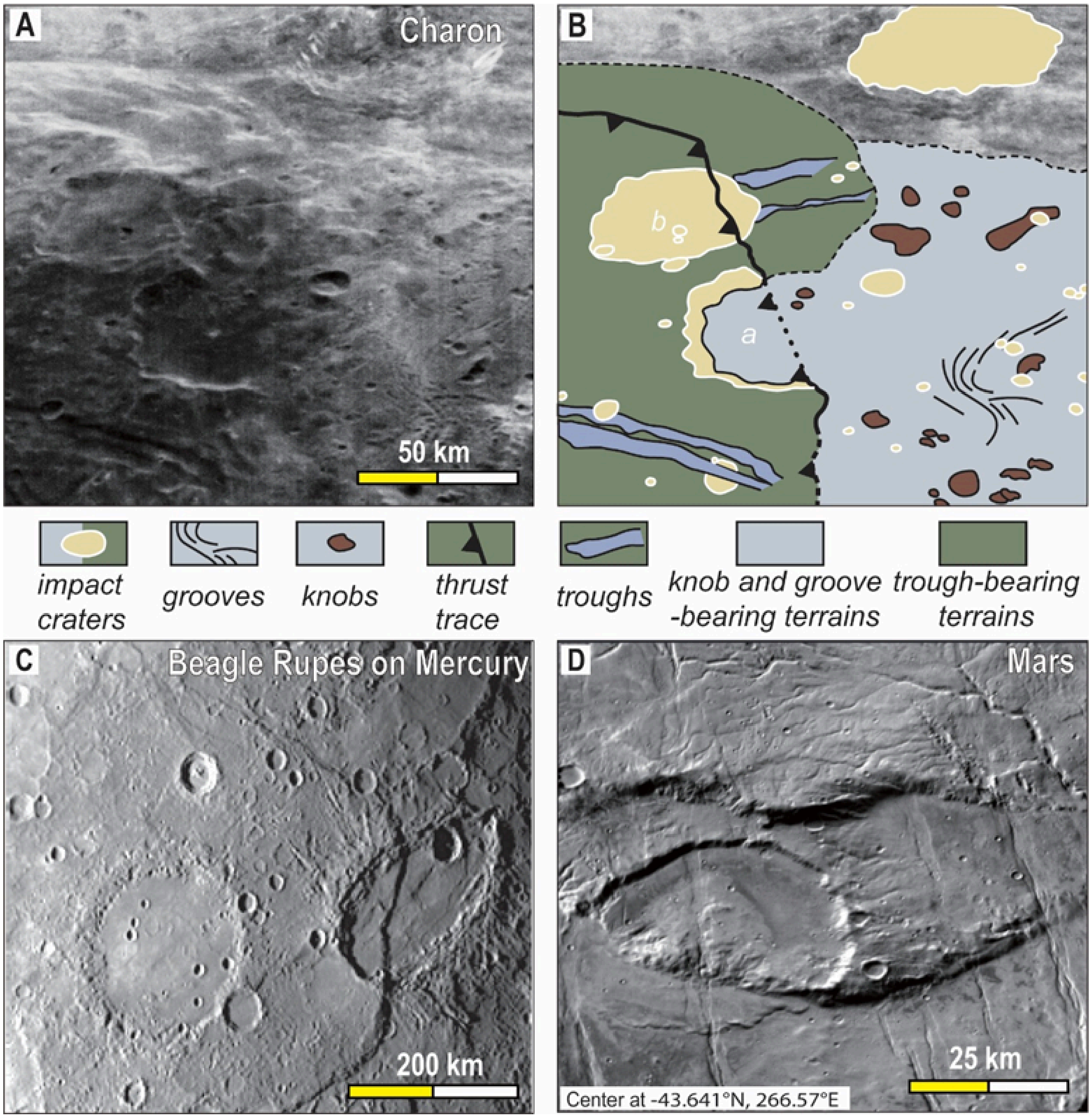

Fig. 3 Comparison of half-crater structure on Charon with planetary analogues. (A) The half-crater landform along arcuate range *I* shown in the highest-resolution LORRI image. (B) Geomorphological map with two modified craters (*a* and *b*) from the landforms shown in (A). Only the western half of the crater remains discernible, with the eastern half missing. The western half of the crater is located in the trough-bearing terrains, while the eastern half is probably buried under the plain materials (knob-bearing terrains). A vertical offset surpassing the crater depth is required for such contrast, which can be generated by thrusting. Crater *b* is a flat-floored crater with a scarp cut across the crater floor, with the left side slightly uplifted. (C) Image showing the Beagle Rupes on Mercury, where a lobate scarp cuts across a crater. Interpreted thrust that produced the lobate scarp landform also elevated the left half of the crater relative to the right half [37]. (D) Half crater along the margin of the Thaumasia Highlands on Mars, with the northern half elevated and preserved, while the southern part is missing, attributed to a compressional tectonic origin [38]. (A) is from PIA10939 in NASA's photojournal database.

[R1C6] Regarding craters a and b, the authors do an excellent job of considering the implications of observations, as well as including detailed comparisons to other worlds, exactly as hoped for in such circumstances. The alternate hypothesis of mantling infill is appropriately considered and noted. A few adjustments in wording however would perhaps strengthen the authors' case. Crater a's missing half actually does have a faint visible remnant, suggesting it was not totally 'obliterated', as described in text. A further alternate hypothesis is that crater a formed on top of a pre-existing division of landforms, and the degraded half retains far less profile because either it was a severe lowland to begin with and never built any sharp eastern wall, or that it was a warmer, more damaged, or more compositionally distinct terrain type that degraded faster.

Thank the reviewer for raising more alternative hypotheses to the observed half crater structure. For the mantling hypothesis, after careful investigation into the landform adjacent to the half crater, we did not see any features that resemble remnant crater rim ridges. However, the further extent of the smooth surface lacking knobs may represent the boundary of the lower half crater. We appreciate the suggestion of considering the landscape-division hypothesis. For the severe low-land case, thrusting will serve as a favorable mechanism to have formed such an elevation change, as discussed in the manuscripts. The thermal-state or composition difference hypotheses are both plausible. We included those alternative interpretations in the text, lines 118 - 121: "*The eastern half of the crater could have been mantled by the resurfacing materials or degraded within significantly warmer or weaker materials, but it still requires a vertical offset greater than the crater depth or a sharp boundary for such a distinct contrast.* ".

[R1C7] Just southeast of crater a on the same candidate scarp, and just outside the southeast edge of the boundary of Fig 3, is a third crater that would seem to also have the same cross-cutting as crater b, with the scarp grazing the edge. Please note if this was considered, and if this cater was omitted from study for simple reasons such as lacking sufficient image resolution. The overall case for the relative timing for craters a and b and tectonic activity is well-justified in the text.

We thank the reviewer for the positive feedback on our interpretation of relative timing for modified craters and tectonic activity. We noticed the crater further southeast of crater *a*. No clear tectonic deformation can be discerned in this crater's floor. Thus, we interpret this crater to postdate the thrust belt rather than getting tectonized as crater *b*. We now added a sentence noting this crater in text lines 136 - 138: "*A similarly sized crater at the southern end of arcuate range I overprints its topography (Fig. 1) and therefore appears to postdate the development of the range.* "

[R1C8] The sentences highlighting that features are unlikely to be the result of any single seismic event, and are almost certainly the result of many events, could be removed to accommodate some length for other clarifications. The possibility of such features from one seismic event ~4.0 Ga is so unlikely that it could safely go without saying.

We thank the reviewer for the suggestion and deleted two sentences in the text from lines 149 - 152.

Section 6:

[R1C9] The authors note that the fit of features indicates limited true polar wander. However, this seems to invert the dependency. It would seem instead that achieving a fit is instead predicated upon a starting assumption that true polar wander is limited.

We thank the reviewer for raising this concern. We agree that limited true polar wander is a fundamental assumption for our fitting work. However, we decided to keep this argument, as we raised the question of whether Charon underwent true polar wander in our introduction section. Besides, 'What's the implication for true polar wander of this work' is a common question that we received in our previous presentations of this work. Thus, to keep the article clear and complete, we want to keep this statement.

[R1C10] For the clause, 'resulted from recession', please clarify, 'orbital recession'?

We thank the reviewer for pointing this out, which caused confusion for the readers. We clarified the recession to be orbital recession in lines 236 - 241 and added text: "*In addition, the alignment of latitudinal-varying tectonic features to the despinning-induced tectonic pattern indicates limited true polar wander [16] of Charon after its despinning process. The larger compressional strain along arcuate range I, closer to the sub-Pluto point, may have partially resulted from orbital recession [31].*"

[R1C11] If results are a full order of magnitude greater than inferred from flexure, please briefly note in text, such as with one sentence, why the flexure results might be so far off?

We thank the reviewer for emphasizing the difference. We think that flexure fitting may capture the lower value during the resurfacing event that formed Vulcan Planitia, where breakup of the primordial icy crust and emplacement of cryo-lava would foreseeably reduce the effective elastic thickness value during that epoch. We added in line 245 - 246: "*The ~2 km estimate from flexural fitting may have captured the thermal anomaly caused by cryovulcanism during global extension.* "

[R1C12] It seems incongruous that for the nomenclature in the Charon literature, 'cold start' is connected to the giant impact hypothesis. For other worlds, 'cold start' is reserved for situations with very slow accretion, heat escape prior to burial, and a lack of any massive thermal event later such as a giant impact. How is a giant impact not a 'warm start', or 'warm reset', relative to even colder/more-quiescent alternates? (e.g., is the 'cold start' of a Charon giant impact actually referring to the 'start' being long before the giant impact occurred? And if so, wouldn't its traces, at least upon shell depth, not be reset by the impact?) While the cited literature likely explains this at length not available here, it would be best to help readers understand this manuscript's final and strongest conclusion, by incorporating just one brief sentence explaining this seeming incongruity in the nomenclature.

We thank the reviewer for pointing out this confusion. The giant impact hypothesis in the Charon literature refers to the hypothesis that Charon was formed in a giant impact onto Pluto, similar to that of the Moon-forming impact. In this hypothesis, Charon accreted from the impact-generated disk around

Pluto, instead of accreting separately in the planetary disk. More information on this can be found in the reference: McKinnon et al., (2021) [21] and Denton et al., (2025) [33].

The aforementioned studies have suggested that the Charon-forming impact should have caused thermal anomaly mainly on Pluto, with a temperature increase around 50 K. In contrast, thermal enhancement on Charon after the impact is estimated to be less than 30 K. Besides, this giant impact event may have postdated the half life of the ^{26}Al, whose decay contributes to the heat budget in the early thermal evolution of planetary bodies. Thus, the giant impact hypothesis is compatible with a cold start for Charon and a warm 'start' for Pluto. To clarify these points, we now revised sentences in line 248 - 254: "*Such a thick ice shell favors a cold initial thermal state ("cold start"), as predicted by the thermal evolution models [19, 20, 32]. This scenario is compatible with the giant impact hypothesis for Charon's origin proposed by [21, 33], in which a giant impact produced a thermal anomaly primarily on Pluto and resulted in the accretion of Charon from relatively cold ejected material. A cold start is further congruent with the need for global contraction to accompany despinning in reproducing the observed tectonic patterns.* ".

Modeling and equations:

[R1C13] The model is well-presented in fully appropriate detail. The model parameters are well presented in table format and the numerical values for those items subject to author choice are well-chosen. Software and data sources are well-described. No concerns with the numerical modeling are noted. Figures 4, 5 and 6 are robust and clear. The method used is novel, by using the terrain positive deformation fit as a fully reasonable approach to constraining subsurface geometry. There is some concern that the fit might be influenced as much by other parameters as the two chosen for primary study, but this is a minor worry, and such multidimensional equation systems must limit fitting somehow, and the relative parameter sensitivities are well enough addressed to make the needed case here.

We thank the reviewer for raising minor concerns about the potential influence of other fault parameters on the deformation pattern. We referred to the study of Peterson et al. (2020) in the application of the Okada model. Here presented is a figure from their paper stating the influence of different fault parameters on the surface deformation. Among all the parameters, displacement is the dominant controller on topographic relief and can be estimated from the relief of the ranges. Fault burial depth (Z_b; or the depth of the fault tip) contributes less compared to the fault dip angle (θ_b) and fault maximum depth (Z_{max}; or fault maximum depth). Tapering is not considered in our study as the planar model already generates decent topographic fits. Thus, fault dip angle (θ_b) and fault maximum depth (Z_{max}) are the two main parameters considered in our modeling. Because the contribution of different parameters in the Okada model has been explicitly studied in previous papers and due to the length limit for the text, we did not include a detailed discussion on the selection of parameter pairs but now add a brief description of selected model parameters.

We now add text in lines 438-440: "*As discussed in [28], besides slip amount that can be estimated from topographic relief, fault dip and fault root depth are the two major parameters that control the configuration of the displacement field.*".

[REDACTED]

Figure R3. Taken from Figure 1 in Peterson et al. (2020). The panels showcase the influence of varying parameters on surface deformation: (a) fault plane geometry, planar vs. listric; (b) fault root depth; (c) displacement along the fault plane; (d) fault dip angle; (e) fault tip depth; (f) tapering of displacement along the fault plane.

Overall, the possibility that Charon is indeed a case of expression of the Voyager-era despinning surface predictions, is a highly valuable study and a possible landmark determination. The authors are commended for a well-presented and logical chain of inferences from limited surface imagery, using a robust model approach, to new constraints on formation scenarios. Again, the only strong concern is just with improving justification of what linear features are chosen or set aside, based on their apparent topography. I look forward to seeing this work with such minor adjustments and then soon seeing its eventual publication.

Thank you again for your constructive comments. We hope that our revisions fully address your concerns and are sufficient for publication in *Nature Communications*.

Reviewer #2 (Remarks to the Author):
This manuscript argues that arcuate positive relief ranges in Charon's Oz Terra are compressional structures formed by thrusting and that their distribution, combined with other tectonic trends, records an early tidal despinning signal. The authors fit two topographic swath profiles with an elastic dislocation thrust model to infer fault geometry and a minimum elastic shell thickness of roughly 30 to 36 km, then convert inferred shortening into about 1 percent equatorial compression and an implied early rotation period near 14 hours. They compare predicted despinning stress provinces to mapped tectonics and invoke an added global contraction component to better match the observed latitude extent of compression. I recommend publication with revisions.

The logic mostly progresses in a clear sequence: mapping and interpretation of the arcuate ranges, mechanical inversion for fault geometry, conversion of shortening to regional strain, conversion of strain to flattening and initial spin, then comparison of predicted stress provinces to observations. The main intellectual jumps occur where local measurements are scaled to global quantities and where additional stress terms are introduced to reconcile mismatches.

We thank the reviewer for their positive and constructive comments, which identified the places for improvement . We agree that a more explicit explanation of our interpretation, along with the rationale for eliminating other mechanisms, will enhance the robustness of our study. We have now added a section in the method part (Uncertainties in elastic modeling and inferred stress estimates) for a discussion on the wider range of elastic thickness values and corresponding stress fields, which predict reasonable outcomes for our interpretation (Fig. S3). We thank the reviewer again for their constructive comments, which improved the robustness of our results and interpretations.

To bridge the gap between local measurements and global quantities, we revised text in the Methods section, lines 492 - 496: "*Here, we assume the fault root depth as the detachment depth and derive a horizontal shortening of ~ 2.9 km accommodated by the thrust. Taking the average spacing of ~ 280 km between arcuate ranges I, II, IV in Fig. 1 as the reference length, this shortening amount corresponds to a regional compressional strain of ~1%.*"

Major comments:
[R2C1] The manuscript makes a plausible thrust interpretation, but it moves quickly from "arcuate positive relief" to "compressional thrusting" without a fully explicit elimination of other mechanisms. The authors do acknowledge normal faulting as less likely in one key place, which helps, but the review would be stronger with a more structured argument for what observations are uniquely diagnostic of thrusting and what observations would be expected under competing hypotheses.
We thank the reviewer for raising this concern. This is a comment that we received from both reviewers. To give a more explicit reasoning on our interpretation, we added a section (Methods: Topographic Analysis and Identification of Candidate Arcuate Ranges) in the method part together with a new figure (Fig. S1) and a new table (Table S1) to explain our criteria that lead to our interpretation. Please see the response for R1C2, presented on pages 3-6.

[R2C2] Non uniqueness of the inversion and the mapping from fault root depth to elastic thickness
Okada style dislocation fits can be non-unique, with trade offs among dip, depth, slip magnitude, and slip distribution. The manuscript reports best fits and uses inferred root depth as a lower bound on elastic thickness, but the text does not always adequately discuss the extent of any non-uniqueness in the solution. In addition, treating the fault root depth as the detachment depth for balanced cross section shortening is a substantive assumption. Mechanical stratification could allow detachments above or below the effective elastic thickness. This does not invalidate the approach, but it should be framed as an estimate with wider uncertainty and supported a discussion of sensitivities and uncertainties in the solution.

Thanks for the suggestion. We acknowledge that we did not include adequate discussion on the non-uniqueness in model fitting and the assumptions for the application of the balanced cross section

method in the previous submission. We now added a section in Method (Uncertainties in elastic modeling and inferred stress estimates) that discusses the estimate of elastic thickness in a wider range. The fits with nRMSE<1.0 in the figure give a range of both fault root depths and fault dip angles. and compared the stress field of a wider range of elastic thickness values (Fig. S3). In addition, we now added a supplementary figure showing the required minimal additional compressional stress as a function of the crustal thickness/radius ratio (t/R). Please see the added section below and the new Figure S3.

8.5. Uncertainties in elastic modeling and inferred stress estimates

We acknowledge that uncertainties exist in our elastic modeling approach used to estimate crustal thickness, which lead to subsequent uncertainties in the inferred despinning-induced stresses and the additional stress required. In this section, we explain the sources of these model estimate uncertainties, justify our modeling choices and the adopted model setup and parameter ranges, and discuss their implications for our conclusions.

First, we use the best-fit values of elastic thickness of Charon's crust from an elastic dislocation model applied to the topographic features from Charon. However, there is uncertainty from the estimate of elastic thickness due to the trade-off between model parameters. For example, fitting topographic profiles via Okada Model show trade-offs between fault dip angles and fault root depth (Fig. 2), where the influence from increased fault dip can be compensated by the increase of fault root depth. The nRMSE values indicate a trend of higher values (worse fits) as fault parameters continue to increase after fault root depth surpassing ~40 km and fault dip surpassing ~35°. Varying the fault root depth by ~ ±5 km around the chosen best fit value of 36 km corresponds to ~ ±0.01 deviation in crust thickness/radius (t/R) ratio. This range results in relatively small latitudinal variation extent of tectonic provinces (less than ±1° in Fig. 4). The uncertainties from the trade-offs between parameters do not cause significant deviation from the best-fit pair case that we presented in Fig. 5.

Second, we assumed the fault root depth as a lower bound for the elastic thickness of the icy crust. However, mechanical stratification may localize detachment along weak layers, allowing elastic thickness to be different from the inferred fault depth. To explore the impact of potential ranges of elastic thickness of Charon's icy crust, we quantify the influence of varying elastic thickness on the extent of predicted tectonic provinces (Fig. 4) and on the minimum additional compressional stress required to match with the observed tectonic configuration (Fig. S3c). Based on previous studies of Charon's interior structure [e.g., 13, 19, 20], we examine elastic thickness layer thickness values ranging from ~ 6 km to 240 km, corresponding to t/R values of 0.01 to 0.4. The lower bound (~ 6 km) is derived from elastic thickness estimates obtained from flexural fits in a previous study [13], and the upper bound (~240 km) corresponds to the inferred total thickness of Charon's icy shell from interior structure models [20].
To illustrate the differences, we present two examples of different elastic thickness (t/R values of 0.1 and 0.2). For t/R = 0.2, the model predicts compressional tectonic province at equatorial area, strike-slip dominant province in the mid-latitude region, and extensional tectonics around the poles (Fig. S3a). An additional 6 MPa compressional stress is needed to match the stress field with observed extent of tectonic features. For t/R = 0.1, no equatorial compressional tectonic province is predicted, where an additional 8 MPa compressional stress is needed (Fig. S3b). The required additional stress within possible Charon crust thickness values increases as elastic crustal thickness decreases (Fig. S3c), from ~2 MPa of a 240-km crust to ~26 MPa of a 6-km crust. Although we did not model porosity reduction during the relevant timescale, these estimates are plausible considering global contraction from subsurface ocean growth. Previous studies have estimated ~ 100 MPa accumulated extensional stress from the subsequent refreezing of the subsurface global ocean that induced global extension [46]. Lastly, there is also uncertainty in determining the latitudinal extent of tectonic features. We present their variations shown by

the colored curves in Fig. S3c, which shows small (< 1.5 MPa) variations in the estimation of required stress.

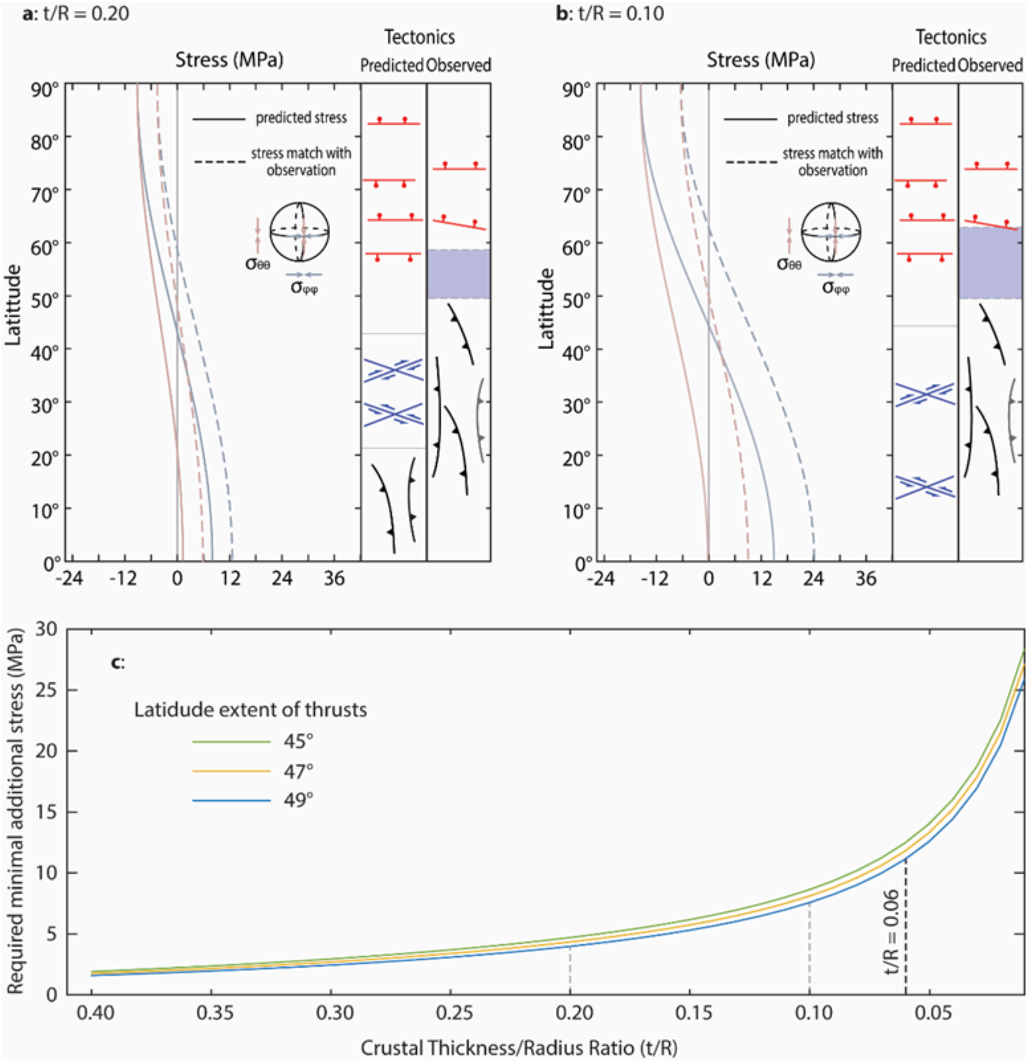

Fig. S3 Calculated stress field at different crustal thickness and corresponding additional stress to match with observation. *Examples of crustal thickness at 120 km (a) and 60 km (b) present the variation in calculated stress (solid curves) and corresponding tectonic patterns. The stress field required to match with the observed extent of tectonic provinces (dashed lines). (c) plot of crustal thickness to radius ratio and required minimum additional stress at different scenarios of thrust distribution, with latitudinal extent to 45° (green), 47° (yellow), and 49° (blue). The crust to radius ratio of 0.20 (grey dash line), 0.10 (grey dash line), and 0.06 (black dash line) correspond to the three cases shown in panel a, b and in Fig. 5.*

[R2C3] To match the observed latitude extent of thrust tectonics, the authors add an additional compressive stress of about 20 MPa attributed to global contraction. This is plausible, but it is introduced primarily to reconcile a mismatch between prediction and observation. The argument would be more convincing if the contraction magnitude were constrained independently, for example via thermal evolution or porosity closure estimates, or if the paper showed that a reasonable range of contraction magnitudes that still yield the correct tectonic province pattern.

To address the concern on the magnitude of required additional compressional stress, we recalculated the stress and updated the value to 12 MPa for a ~40 km elastic crust in text line 202 - 204. We also

systematically explored the specific stress magnitude for a range of crustal thickness values (Fig. S3c), where we also included the variation of the latitudinal extent of tectonic patterns.

We agree with the reviewer that independent constraints from thermal evolution and porosity closure estimates will make our argument more convincing. Presented below (Fig. R4) is Figure 1 in Bierson and Nimmo (2018) on the thermal evolution of Pluto and Charon. This figure exhibits a nominal model output for the 1-D thermo evolution of both Pluto (top panel) and Charon (bottom panel). For Charon, the modeling work exhibits a 10s of kilometers decrease in radius due to the porosity reduction and the growth of a subsurface ocean (the black envelope in the figure). The thickness of the subsurface ocean reaches up to around 50 km in their modeling work. The numerical modeling works suggest larger values of both radius decrease and subsurface ocean thickness compared to the required 2-km radius change in our study.

[*REDACTED*]

Figure R4. Taken from Figure 1 in Bierson and Nimmo (2018). The lower panel showcases a plausible 1-D thermo-evolution model for Charon through time that ends up at Charon's current state. The temperature is color-coded, with dashed temperature contours. The brown line represents the ice-rock boundary. Black pocket represents a subsurface ocean. A pink-dashed curve delineates the depth of the porous layer.

Set porosity reduction apart, we compare the required additional compressional stress to the estimation of accumulated extensional stress. From both the prediction of thermal evolution (e.g., Bierson and Nimmo, 2018) and estimation of extensional strain (e.g., Spencer et al., 2021), the accumulated extensional stress from the refreeze of the subsurface ocean is ~100 MPa. The growth of such a subsurface ocean can provide a strain budget sufficient to generate the required compressional stress. We now added related text in the section: Uncertainties in elastic modeling and inferred stress estimates. Please see page 18.

[R2C4] Stress province comparison would benefit from more explicit mapping uncertainty
The discussion notes discrepancies such as the absence of predicted mid latitude strike slip systems. This is a useful observation. It would be strengthened by clarifying whether strike slip absence is a data coverage limitation, a mapping completeness issue, or a true geologic absence that challenges the model assumptions.

We thank the reviewer for the suggestion. In section 5: Despinning origin for the thrusts, we discuss briefly the missing mid-latitude strike-slip faults and propose two plausible hypotheses. Lines 210 - 216: "*The strike-slip systems may have developed in the mid-latitude region of Oz Terra, but are indiscernible due to a lack of significant vertical displacements across strike-slip faults, while the surface ruptures are obscured by space weathering. Alternatively, the strike-slip systems can be incorporated into the thrust systems, where the arcuate ranges are produced by transpressional faults.*"

In the last paragraph of section 6, we previously discussed that the missing strike-slip systems could have also been due to the flaws in the failure criteria (Anderson's fault theory) adopted in Melosh's model. Anderson's fault theory predicts the fault types based on the relative magnitudes of principal stresses. However, in a tensile regime, instead of having the deviatoric stress accumulate to reach the Mohr strength envelope, the tensile strength of the materials may be exceeded first. The resulting rock failure will become mode-I fractures, instead of thrusts or normal faults. On terrestrial planets, deeper in the crust, the exerted gravitational force from overlying crust sections helps to avoid this tensile regime (see Klimzack et al., (2015)[36]). In contrast, the low gravity and density of Charon do not favor such a solution. We proposed to work on this problem in our future studies, in lines 272 - 277: "*In particular, our criteria for identifying tectonic patterns follow Anderson's theory instead of incorporating a more realistic brittle failure mechanism such as the Griffith criterion [36]. Future work could integrate failure envelopes or stress-dependent rheological models to better capture the mechanical behavior of the ice shell and improve predictions of fault style and distribution on Charon.* "

REVIEWER COMMENTS

Reviewer #1 (Remarks to the Author):

This work is of extremely high quality and is strongly recommended for publication with minor revisions. The text is free from nearly all forms of minor faults and is proofed to a very high degree. The approach is novel and of unique scientific value. The methods are solid, well-presented, and make a convincing case from observations to conclusions. All figures are appropriate, accurate, and well-explained. The result is timely and important, as a possible first case of despinning evidence preserved on an ancient global terrain. A few relatively minor concerns are noted below, mainly with the selection and justification of inputs to the model, and not the model itself nor its final interpretation. The concerns noted are similar to issues encountered by many geology papers, and not unique to this work by any means. Therefore, it is expected to be resolved with ordinary adjustments to the wording.

The authors conclude with a signal (in shell thickness best-fit) that is sufficiently strong, that even if it were off by say a factor of 2-3, it would still represent a reasonable preference for the stated conclusions, in particular of a colder start than other reports have shown. This strong signal in the results helps to mitigate any worries for minor numerical assumptions.

Geologic interpretation on Charon is of course challenged by limited data, and the authors have done an excellent job of making the most out of the resolution, spatial coverage, and single-angle-of-illumination available.

Specific Notes:

Figure 1: The sentence noting the meaning of grey arrows is repeated in the caption. One instance can be removed.

Thank you for the careful proofread. We have removed the repeated parts.

A primary concern for this work regards the selection of features to be considered as candidate faults. Figure 1 is open to considerable differences in geologic interpretation, particularly as to what landform edges are included for study. It is naturally a longstanding and universal concern

for geology papers, that human subjective interpretation of complex landforms has always been an issue. (Ideally someday in the future, machine-learning based edge-detection algorithms might be standardized enough to allow less subjective determination of what is declared an arcuate/lobate feature and what is not. However, this manuscript must not be held to any higher standard of proof than all previous literature, and thus human subjectivity remains the widespread baseline for such works.)

In effect this is asking the authors to not only justify the positive selection of candidate features declared to be arcuate scarps of any confidence level, but also to justify the negative side as well. This means, briefly explain why visibly-apparent semi-arcuate or semi-linear features visible in Fig. 1 that are not labeled with grey or white arrows, are deemed by the authors as not being candidates. Many possible reasons come to mind (e.g. features already studied in other literature as having a distinctly different origin, which is perhaps not in conflict with the study here). But it is best for the authors to state their own case-by-case logic.

In particular, the following un-labeled features should be briefly explained for their non-selection, because they appear to go against the hypothesis of north-south orientation predominance:

We thank the reviewer for raising this concern. We acknowledge that ambiguity is inevitable in the process of geologic mapping. Never the less, the limited data in planetary geology made it even harder to interpret structures. Here, we present a table comparing the landforms that we define as arcuate ranges and the scarps referring to the terrestrial-based tectono-geomorphology.

**Landforms**	**Arcuate Ranges**	**Scarps**
**Characteristic topographic wavelengths**	~30 – 50 km	~10 km
**Maximum slope**	< [illegible]	> [illegible]
**Map view traces**	arcuate	straighter and in segments
**Elevation changes**	lower elevation towards convex direction	lower elevation towards concave direction
**Ridge crest morphology**	Broader rounded ridge tops	sharp ridge crest lines
**spacing**	more isolated	can appear in pairs as bounding scarps of grabens

Inducing structures	Underlying thrusts	Normal faults

- The lower righthand quadrant of Fig. 1 is filled with multiple parallel east-west linear elevation change features.

Sharp ridge crests, planar slopes (potentially normal fault scarps), mostly concave towards lower elevation within each segment.

- Starting from the topographic local minimum of the deep blue circle in the upper left, trending diagonally to the upper right corner of Fig. 1, can be interpreted as a lengthy linear feature of mostly east-west orientation.

Broad grabens, no sharp bounding scarps.

- Likewise, an east-west feature (almost exactly parallel to that noted just above) spanning all of Fig. 1 appears to start from the lower lefthand corner, traversing to the midpoint of the righthand edge (with some interruption). Again, please note in the text why this feature, or segments of it, are not included for study here.

Sharp ridge crests, planar slopes (potentially normal fault scarps), trough morphology.

- Fig. 1 and Oz Terra in general could be interpreted as being broken up into several large massifs or elevated landform blocks, about 6-10 in total, each roughly rectangular. But by such interpretation, edges visibly seem to exist on all sides, north, south, east and west combined.

We acknowledge the interpretation proposed by Beyer et al., 2017 paper. However, in this interpretation, the surface morphology differs in adjacent blocks, the 'edges' also vary in morphologic characteristics (e.g. slope, crest line, map view traces).

-The northernmost 'hook' of the scarp containing profile B-B', seems to have about equal visible confidence as an alternate path trending to the west from the point where B-B' crosses. The alternate path would appear less like a north-south overall pattern, and more like a perimeter to a regional upland massif block, (as the scarp containing A-A' already appears). Please justify this and similar choices in the text.

in optical images, the traces appear to extend towards northeast rather than going northwest. Besides, we can see another set of linear features trending NW that may have caused the elevation change that reviewer pointed out. However, these features are beyond the coverage of higher-quality digital elevation model.

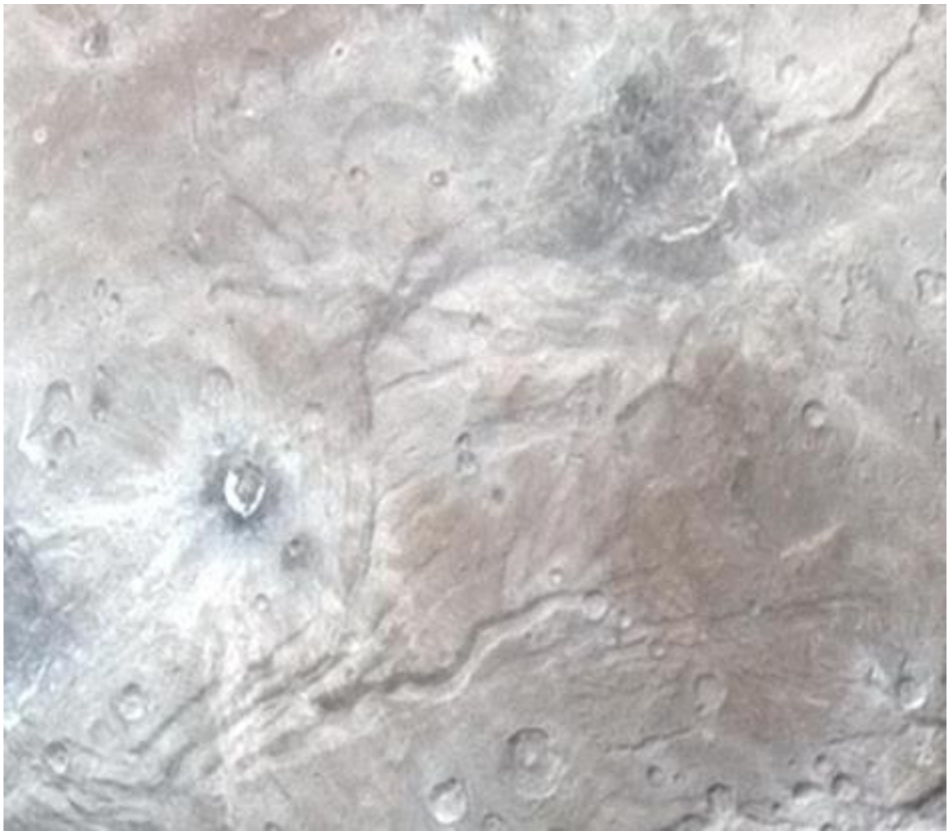

- At about 50 deg north and between 15 deg W and 0 deg, there exists a linear feature which likewise appears to be lobate in form, yet is mainly east-west in orientation. This feature is clearly not a remnant crater rim. Again, it appears as the lower perimeter of an upland massif. Please note in text why this feature is decided not a candidate for study here.

This lobate feature appear to be a northern bounding scarp of a broad east-west trending trough. Or, in Beyer et al., 2017, a southern bounding scarp of a 'crustal block'. Thus, less likely to be an arcuate range.

The authors are encouraged to label the noted scarps, so that they can easily be referred to individually by readers.

The authors are urged to change language regarding the use of an elastic model in this work. The manuscript first states that craters a and b appear to have 'negligible' relaxation. However, again, this term is open to considerable subjective interpretation, and both of these craters could just as well be described has having a degree of relaxation about halfway from appearing fresh, to the opposite extreme of becoming largely erased by relaxation alone (or thus appearing 'halfway relaxed' or 'at a medium degree or relaxation' or 'at a degree of relaxation that is somewhere between end-member extremes'. This is partly a problem of having limited immediate comparisons to work from. The authors are again commended for doing the best one can with limited image data.) This wording would be of little concern, except that it is used

later to justify the elastic model choice. I would urge the authors to change how this is achieved:
- A primary reason for using an elastic model, is simply that such studies must begin somewhere, and an elastic model is almost always the appropriate first choice, especially in the outer solar system. In fact, there is high value for studies to begin by publishing elastic results, to serve as an invaluable baseline against which any non-elastic future investigations must be compared.
- If the relaxation of the total field of Oz Terra were felt by any reader to be 'moderate' instead of 'negligible', the elastic choice and cascading results would thus seem weakened to that reader.
- There are many reasons why a non-elastic model should be tested by future work (after this work is published). Among them are the point that the basal temperature of the ice layer is substantially higher than the surface temperature, with brittle elastic behavior often apparent at the surface, including the ability to support sharp topography long-term, yet the majority of the ice thickness can still best be considered ductile. (Certainly this work does not need to include such an upgrade, yet the authors are of course encouraged to consider this for their own future research directions.)
- As the viscous regime is also a function of stress magnitude, it thus can vary with time, depth and laterally within a single fault system. Ideally, the authors are urged to include in the text, the anticipated creep regime based on both thermal state and deviatoric stress, particularly for conditions expected around ~4.0 Ga on Charon. This is expected to help justify the elastic assumption used.
- Therefore in total, wording is recommend akin to "an elastic model is assumed for investigation based on surface relaxation that is not severe, as well as the literature standard of utilizing an elastic baseline in the absence of pronounced or obvious viscous requirements. Future work will no doubt test this assumption using increasingly detailed viscoelastic models including temporal-spatial variation of controlling parameters."

Akin to the concern for subjective case selection for scarps overall, is the concern for profile selection of A-A' and B-B'. Please briefly note why these two profiles are chosen for reporting, above all other possible slices. (E.g. it seems A-A' was chosen because it contains a topographic highpoint, for maybe better signal to noise?) B-B' seems moderately surprising, because it is not drawn exactly perpendicular to the local scarp proposed orientation and crosses a possible secondary ridge tail in the background terrain. This however may be a purposeful demonstration that the model can match even off-perpendicular and off-ideal slices, and if this was done for this reason, briefly state so.

We thank the reviewer for raising the problem of selection, which will improve this manuscript. We select these two profiles mainly because of better mosaic resolution and DEM quality at these two locations. Please see the attached Schenk 2018 figure 1 for details.

The intended location of profile B-B' is to avoid the crater in the hinterland and east-trending troughs in the foreland. In the application of the Okada model and the balanced cross-section method, it is OK to be not exactly perpendicular. Though in such scenario, the kinematics will become apparent slip instead of true slip. However, the thrust belt where the profile B-B' crossed appears in segments, as you can see the positive-topography belt consists of 2-3 segments of eastward-convex arcs and overprinted by the troughs in its southern section. In this case, the orientation of 'exact' perpendicular is hard to assess. The error caused by obliqueness can be quantified as $\cos(\gamma)$, where γ is the deviation angle from perpendicular direction.

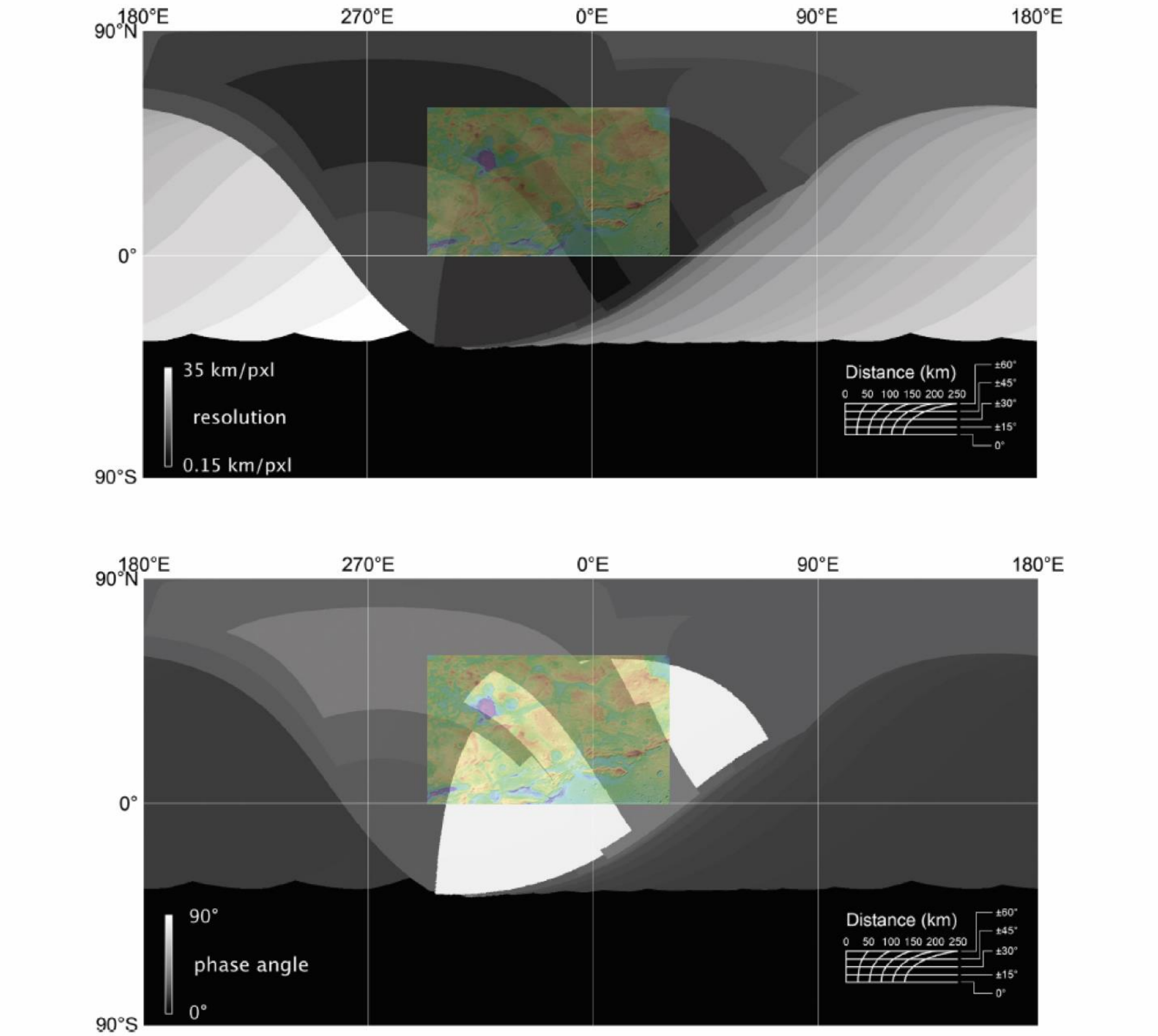

Fig. 2. Global maps illustrating the pixel scale, phase angle, incidence angle, emission angle associated with the panchromatic base map of Charon in Fig. 1. Global

Ideally, if the authors have tested further fits beyond A-A’ and B-B’, this may be included in the manuscript without compromising total length, simply by noting, if true, something like “Numerous additional profiles were tested for fit and found qualitatively indistinguishable to those highlighted in Fig. 2.”

We thank the reviewer for suggesting including more profiles. We did attempt to model other arcuate ranges. However, the two profiles that presented are the most characteristic range profiles. Others may have been modified by other processes or not in identical asymmetric ridge shape.

Fig. 3 labeling and caption: It seems notable that what is referred to as ‘knob-bearing terrain’ is also ‘groove-bearing terrain’, as none of the labeled and visible grooves appear in the trough-bearing terrain. The authors may find it useful to revise to reflect that grooves, maybe more than knobs, set this area apart.

We thank the reviewer for this comment. We updated the legend to include groove-bearing for the specific landform unit.

Regarding craters a and b, the authors do an excellent job of considering the implications of observations, as well as including detailed comparisons to other worlds, exactly as hoped for in such circumstances. The alternate hypothesis of mantling infill is appropriately considered and noted. A few adjustments in wording however would perhaps strengthen the authors’ case. Crater a’s missing half actually does have a faint visible remnant, suggesting it was not totally ‘obliterated’, as described in text. A further alternate hypothesis is that crater a formed on top of a pre-existing division of landforms, and the degraded half retains far less profile because either it was a severe lowland to begin with and never built any sharp eastern wall, or that it was a warmer, more damaged, or more compositionally distinct terrain type that degraded faster.

Thank the reviewer for raising more alternative hypotheses to the observed half crater structure! For the mantling hypothesis, after careful investigation into the landform adjacent to the half crater, we did not see any features that resemble remnant crater rim ridges. However, the further extent of smooth surface lacking knobs may represent the boundary of the lower half crater. We appreciate the suggestion of considering the landscape-division hypothesis. For the severe low-land case, thrusting will serve as a favorable mechanism to have formed such elevation change, as discussed in the manuscripts. The thermal-state or composition difference hypotheses are both plausible. We included them in the text.

Just southeast of crater a on the same candidate scarp, and just outside the southeast edge of the boundary of Fig 3, is a third crater that would seem to also have the same cross-cutting as crater b, with the scarp grazing the edge. Please note if this was considered, and if this cater was omitted from study for simple reasons such as lacking sufficient image resolution.

Discussed in Chen and Yin 2022 paper. The third one postdates the thrust belt rather than syn-post as crater b.

The overall case for the relative timing for craters a and b and tectonic activity is well-justified in the text.

The sentences highlighting that features are unlikely to be the result of any single seismic event, and are almost certainly the result of many events, could be removed to accommodate some length for other clarifications. The possibility of such features from one seismic event ~4.0 Ga is so unlikely that it could safely go without saying.

We thank the reviewer for the suggestion and deleted two sentences.

Section 6:
The authors note that the fit of features indicates limited true polar wander. However, this seems to invert the dependency. It would seem instead that achieving a fit is instead predicated upon a starting assumption that true polar wander is limited.

We thank the reviewer for raising this concern. However, we decided to keep this argument, as ‘implication for true polar wander’ is a common question that we receive after our informal presentations of this work.

For the clause, ‘resulted from recession’, please clarify, ‘orbital recession’?

Thanks for pointing this out, which caused confusion for the readers. We clarified the recession to be orbital recession in line 218.

If results are a full order of magnitude greater than inferred from flexure, please briefly note in text, such as with one sentence, why the flexure results might be so far off?

Flexure fitting may capture the lower value during the resurfacing event that formed Vulcan Planitia, where breakup of the primordial icy crust and emplacement of cryo-lava would foreseeably reduce the effective elastic thickness value. Added in line 225.

It seems incongruous that for the nomenclature in the Charon literature, 'cold start' is connected to the giant impact hypothesis. For other worlds, 'cold start' is reserved for situations with very slow accretion, heat escape prior to burial, and a lack of any massive thermal event later such as a giant impact. How is a giant impact not a 'warm start', or 'warm reset', relative to even colder/more-quiescent alternates? (e.g., is the 'cold start' of a Charon giant impact actually referring to the 'start' being long before the giant impact occurred? And if so, wouldn't its traces, at least upon shell depth, not be reset by the impact?) While the cited literature likely explains this at length not available here, it would be best to help readers understand this manuscript's final and strongest conclusion, by incorporating just one brief sentence explaining this seeming incongruity in the nomenclature.

We thank the reviewer for pointing out this confusion. The giant impact hypothesis in the Charon literature refers to the hypothesis that Charon was formed in a giant impact onto Pluto, similar to that of the Moon-forming impact. In this hypothesis, Charon accreted from the impact-generated disk around Pluto instead of accreted separately in the planetary disk. More information on this can be found in the reference: McKinnon et al., 2020 chapter and Denton et al., 2025 paper.

The Charon-forming impact should have caused thermal anomaly mainly on Pluto, with a temperature increase around 50 K. In contrast, thermal enhancement on Charon after the impact is estimated to be less than 30 K. Besides, this giant impact event may have postdated the half life of the ^{26}Al, whose decay contributes to the heat budget in the early thermal evolution of planetary bodies. Thus, the giant impact hypothesis here suggests a cold start for Charon and a warm 'start' for Pluto.

We added one sentence in line 229.

Modeling and equations:
The model is well-presented in fully appropriate detail. The model parameters are well

presented in table format and the numerical values for those items subject to author choice are well-chosen. Software and data sources are well-described. No concerns with the numerical modeling are noted. Figures 4, 5 and 6 are robust and clear. The method used is novel, by using the terrain positive deformation fit as a fully reasonable approach to constraining subsurface geometry. There is some concern that the fit might be influenced as much by other parameters as the two chosen for primary study, but this is a minor worry, and such multidimensional equation systems must limit fitting somehow, and the relative parameter sensitivities are well enough addressed to make the needed case here.

We thank the reviewer for raising minor concerns on the potential influence of other fault parameters on to the deformation pattern. We referred to the study of Peterson et al., 2020 paper in the application of the Okada model. Here presented is a figure from their paper stating the influence of different fault parameters on the surface deformation. Among all the parameters, displacement is the dominant controller on topographic relief and can be estimated from the relief of the ranges. Fault burial depth (Z_b; or the depth of the fault tip) contribute less compared to the fault dip angle (θ_b) and fault maximum depth (Z_{max}; or fault maximum depth). Tapering is not considered in our study as the planar model already generates decent topographic fits. Thus, fault dip angle (θ_b) and fault maximum depth (Z_{max}) are the two main parameters considered in our modeling.

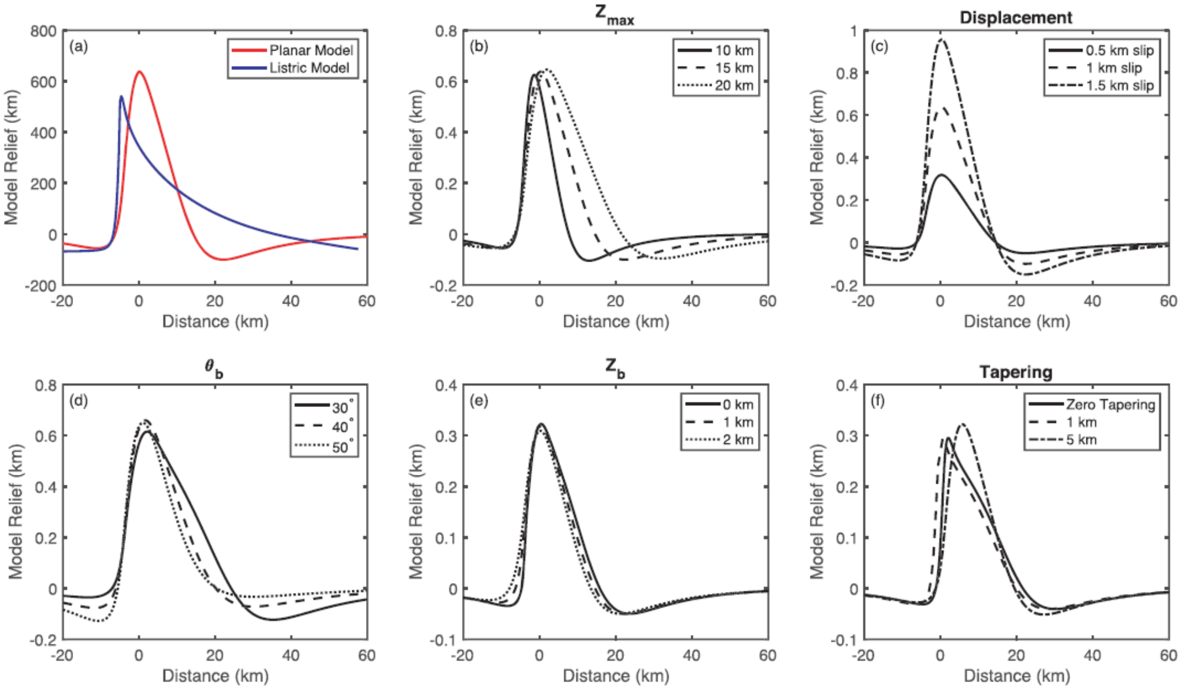

Overall, the possibility that Charon is indeed a case of expression of the Voyager-era despinning

surface predictions, is a highly valuable study and a possible landmark determination. The authors are commended for a well-presented and logical chain of inferences from limited surface imagery, using a robust model approach, to new constraints on formation scenarios. Again, the only strong concern is just with improving justification of what linear features are chosen or set aside, based on their apparent topography. I look forward to seeing this work with such minor adjustments and then soon seeing its eventual publication.

Reviewer #2 (Remarks to the Author):

This manuscript argues that arcuate positive relief ranges in Charon's Oz Terra are compressional structures formed by thrusting and that their distribution, combined with other tectonic trends, records an early tidal despinning signal. The authors fit two topographic swath profiles with an elastic dislocation thrust model to infer fault geometry and a minimum elastic shell thickness of roughly 30 to 36 km, then convert inferred shortening into about 1 percent equatorial compression and an implied early rotation period near 14 hours. They compare predicted despinning stress provinces to mapped tectonics and invoke an added global contraction component to better match the observed latitude extent of compression. I recommend publication with revisions.

The logic mostly progresses in a clear sequence: mapping and interpretation of the arcuate ranges, mechanical inversion for fault geometry, conversion of shortening to regional strain, conversion of strain to flattening and initial spin, then comparison of predicted stress provinces to observations. The main intellectual jumps occur where local measurements are scaled to global quantities and where additional stress terms are introduced to reconcile mismatches.

Major comments:
The manuscript makes a plausible thrust interpretation, but it moves quickly from "arcuate positive relief" to "compressional thrusting" without a fully explicit elimination of other mechanisms. The authors do acknowledge normal faulting as less likely in one key place, which helps, but the review would be stronger with a more structured argument for what observations are uniquely diagnostic of thrusting and what observations would be expected under competing hypotheses.

We thank the reviewer for raising this concern. We acknowledge that ambiguity is inevitable in the process of geologic mapping. Never the less, the limited data in planetary geology made it

even harder to interpret structures. Here, we present a table comparing the landforms that we define as arcuate ranges and the scarps referring to the terrestrial-based tectono-geomorphology.

**Landforms**	**Arcuate Ranges**	**Scarps**
**Characteristic topographic wavelengths**	~30 – 50 km	~10 km
**Map view traces**	arcuate	straighter and in segments
**Elevation changes**	lower elevation towards convex direction	lower elevation towards concave direction
**Ridge crest morphology**	Broader rounded ridge tops	sharp ridge crest lines
**spacing**	more isolated	can appear in pairs as bounding scarps of grabens
**Inducing structures**	Underlying thrusts	Normal faults

Non uniqueness of the inversion and the mapping from fault root depth to elastic thickness Okada style dislocation fits can be non-unique, with trade offs among dip, depth, slip magnitude, and slip distribution. The manuscript reports best fits and uses inferred root depth as a lower bound on elastic thickness, but the text does not always adequately discuss the extent of any non-uniqueness in the solution. In addition, treating the fault root depth as the detachment depth for balanced cross section shortening is a substantive assumption. Mechanical stratification could allow detachments above or below the effective elastic thickness. This does not invalidate the approach, but it should be framed as an estimate with wider uncertainty and supported a discussion of sensitivities and uncertainties in the solution.

Thanks for raising this concern! We added a supplementary figure showing the required minimal additional compressional stress as a function of the crustal thickness/radius ratio (t/R). The better-quality fits in the figure give a range of both fault root depths and fault dip angles.

To match the observed latitude extent of thrust tectonics, the authors add an additional compressive stress of about 20 MPa attributed to global contraction. This is plausible, but it is introduced primarily to reconcile a mismatch between prediction and observation. The argument would be more convincing if the contraction magnitude were constrained independently, for example via thermal evolution or porosity closure estimates, or if the paper

showed that a reasonable range of contraction magnitudes that still yield the correct tectonic province pattern.

We agree with the reviewer that independent constraints from thermal evolution and porosity closure estimates will make our argument more convincing. Presented below is the figure 1 in Bierson and Nimmo's 2018 paper on thermal evolution of Pluto and Charon. This figure exhibits a nominal model output for the 1-D thermo evolution of both Pluto (top panel) and Charon (bottom panel). For Charon, the modeling work exhibits a 10s of kilometer decrease in radius due to the porosity reduction and the growth of a subsurface ocean (the black envelope in the figure). The thickness of the subsurface ocean reaches up to around 50 km in their modeling work. The numerical modeling works suggests larger values of both radius decrease and subsurface ocean thickness compared to required 2-km radius change in our study.

[*REDACTED*]

Stress province comparison would benefit from more explicit mapping uncertainty
The discussion notes discrepancies such as the absence of predicted mid latitude strike slip

systems. This is a useful observation. It would be strengthened by clarifying whether strike slip absence is a data coverage limitation, a mapping completeness issue, or a true geologic absence that challenges the model assumptions.

We thank the reviewer for the suggestion. In section 5: Despinning origin for the thrusts, we discuss briefly about the missing mid-latitude strike-slip faults and proposed two plausible hypotheses. '*The strike-slip systems may have developed in the mid-latitude region of Oz Terra, but are indiscernible due to a lack of significant vertical displacements across strike-slip faults, while the surface ruptures are obscured by space weathering. Alternatively, the strike-slip systems can be incorporated into the thrust systems, where the arcuate ranges are produced by transpressional faults.*'

In the last paragraph of section 6, we discussed that the missing strike-slip systems could have also been due to the flaws in Melosh's model. As the Anderson's fault theory predicts the fault types based on the relative magnitudes of principal stresses, which does not apply to certain extensional regime. On terrestrial planets, deeper in the crust, the exerted gravitational force from overlying crust sections help to avoid this issue. However, the low gravity and density of Charon do not favor such solution. We proposed to work on this problem in our future studies.
